# ARID1A-deficiency in urothelial bladder cancer: No predictive biomarker for EZH2-inhibitor treatment response?

**DOI:** 10.1371/journal.pone.0202965

**Published:** 2018-08-23

**Authors:** Stefan Garczyk, Ursula Schneider, Isabella Lurje, Katharina Becker, Thomas A. Vögeli, Nadine T. Gaisa, Ruth Knüchel

**Affiliations:** 1 Uropathology Group, Institute of Pathology, University Hospital RWTH Aachen, Aachen, Germany; 2 Department of Urology, University Hospital RWTH Aachen, Aachen, Germany; Centro Nacional de Investigaciones Oncologicas, SPAIN

## Abstract

Bladder cancer therapy relies on aggressive treatments highlighting the need for new, targeted therapies with reduced side effects. SWI/SNF complexes are mutated in ~20% across human cancers and dependency of SWI/SNF-deficient tumors on EZH2 has been uncovered recently. To systematically dissect the frequency of genetic alterations in SWI/SNF complexes potentially contributing to their inactivation, mutations and copy number variations in 25 SWI/SNF subunit genes were analyzed making use of publicly available sequencing data for 408 muscle-invasive bladder carcinoma samples. *ARID1A* truncating mutations were identified as the by far most common alterations of SWI/SNF complexes in urothelial bladder cancer. As current ARID1A protein expression data in bladder cancer are inconsistent and incomplete we examined if the frequency of truncating *ARID1A* mutations translates into a similar frequency of cases showing ARID1A protein loss. We applied a validated ARID1A antibody conducting a comprehensive immunohistochemistry-based expression analysis in urothelial bladder cancer (n = 362) including carcinoma *in situ* (CIS) cases. While observing increased median ARID1A protein levels in all carcinoma subgroups compared to normal urothelial controls (n = 21), the percentage of cases showing ARID1A protein loss was positively correlated with increasing stage and grade culminating in a rate of 14.1% in muscle-invasive disease. ARID1A-depletion did neither increase EZH2 protein or trimethylated H3K27 levels *in vitro* nor did ARID1A expression correlate with EZH2 or H3K27me3 amounts in human bladder carcinomas. Importantly, ARID1A-deficiency was neither associated with enhanced sensitivity towards inhibition of EZH2 enzymatic activity nor depletion of EZH2 protein. In summary, *ARID1A* truncating mutations, potentially translating into ARID1A protein loss in a subset of high-grade bladder cancers, are the most common SWI/SNF genetic alterations in bladder cancer. Our data do not support ARID1A-deficiency as predictive biomarker for EZH2-inhibitor treatment response in bladder cancer underlining the need for future bladder cancer-specific, drug screens for successfull discovery of ARID1A-deficiency-based targeted drugs.

## Introduction

An estimated number of 380,000 new cases per year worldwide make bladder cancer the most common malignancy of the urinary tract [1]. More than 90% of all bladder tumors diagnosed in Europe and North America are urothelial carcinomas [2]. The majority of bladder cancers present as non-muscle-invasive, low-grade papillary carcinomas, characterized by an excellent prognosis, while muscle-invasive bladder cancer (MIBC) is associated with an unfavorable outcome [2]. Most MIBCs arise via carcinoma *in situ* (CIS), a flat high-grade lesion associated with *TP53* mutations accounting for ~10% of all bladder tumors diagnosed [3]. Current disease management for bladder cancer mainly relies on aggressive treatments, i.e. BCG-based immunotherapy or chemotherapy in addition to surgery depending on stage and grade of the disease [4,5] highlighting the need for effective targeted therapies with reduced side effects.

Genes encoding subunits of the SWI/SNF (Switch/Sucrose Non-Fermentable) nucleosome remodeling complexes have been reported to be mutated in approximately 20% of all human cancers and functional/mechanistic studies support their role as tumor suppressors [6,7]. SWI/SNF complexes are thought to remodel the nucleosomal architecture of the DNA in an ATP-dependent fashion thereby regulating transcription of cell-cycle-associated genes such as *MYC*, *CCNE1* and *CDKN1A* [8–10]. Moreover, a role for SWI/SNF complexes in various DNA repair types including DNA double-strand break (DSB) repair has been revealed [11,12]. The complexes are diverse and consist of >10 subunits of evolutionary conserved core and variant subunits per complex and these are encoded by 29 genes [13]. Two major subclasses of SWI/SNF complexes are known, the BRG1-associated factor (BAF) and the polybromo BRG1-associated factor (PBAF) SWI/SNF complexes [14]. ARID1A, a subunit enabling sequence-unspecific DNA-binding of BAF complexes [14], is the most commonly altered SWI/SNF subunit across all human cancers [15] and a potential tumor suppressor protein [7,10].

Numerous studies have uncovered a genetic antagonism between SWI/SNF and Polycomb repressive complex (PRC) genes [16–19]. Polycomb group (PcG) proteins assemble to PRCs, with PRC1 and PRC2 being the two best characterized among them. The PRC2 catalyzes the tri-methylation of lysine 27 of histone H3 (H3K27me3) in turn leading to recruitment of PRC1 and establishment of a repressive chromatin state at target gene promoters. The PRC2 consists of one of the two catalytic subunits enhancer of zeste homologue 1 (EZH1) or 2 (EZH2), mediating the histone methyltransferase activity of the complex, and at least two other core components, suppressor of zeste 12 (SUZ12) and embryonic ectoderm development (EED) [20]. Kia *et al*. demonstrated that reintroduction of the core SWI/SNF subunit SNF5 into a SNF5-deficient tumor cell line results in eviction of PRC1 and PRC2 from the *P16INK4A* locus leading to activation of p16 expression [18]. Wilson and colleagues observed that loss of SMARCB1 (SNF5) triggers EZH2 expression, broad H3K27-trimethylation, subsequent repression of Polycomb target genes finally resulting in tumor formation. Importantly, SNF5-driven tumorigenesis could be blocked by inactivation of EZH2 [17]. Moreover, a synthetic lethality relationship between other SWI/SNF components including ARID1A and EZH2 has been revealed in several tumor entities [21,22] but the potential of this concept for urothelial bladder cancer therapy is not known.

In order to systematically analyze the potential applicability of treatments based on SWI/SNF-deficiency—including dependency on EZH2—to bladder cancer, we comprehensively dissected the frequency of inactivating alterations in known SWI/SNF subunit genes in bladder carcinomas revealing *ARID1A* truncating mutations as the most common inactivating events in SWI/SNF complexes. As current ARID1A protein expression data for bladder cancer are inconsistent and incomplete, a comprehensive immunohistochemistry (IHC)-based ARID1A protein expression analysis including carcinoma *in situ* (CIS) cases was conducted for the first time. Moreover, direct evidence of ARID1A’s tumor suppressor capability in urothelial bladder cancer cells is scarce and we thus analyzed the effect of ARID1A protein loss on growth of urothelial cells as well as potential underlying mechanisms. Finally, we investigated the putative dependence of ARID1A-depleted and ARID1A-mutated urothelial cells on EZH2 using *in vitro* models for bladder cancer. So far, our data do neither support a genetic antagonism between ARID1A and EZH2 nor ARID1A-deficiency as predictive biomarker for EZH2-inhibitor treatment response in bladder cancer, underscoring the need for future bladder cancer-specific, high-throughput drug screens for successfull discovery of ARID1A-deficiency-based targeted drugs.

## Materials and methods

### Mutational analysis

Genetic alterations (mutations and copy number variations (CNVs)) of 25 SWI/SNF subunit genes were analyzed in two TCGA cohorts,one comprising 408 [23] and the other 127 muscle-invasive bladder cancer samples [24] using cBioPortal (http://www.cbioportal.org/) [25]. Mutual exclusivity and co-occurrence analysis of genetic alterations in *ARID1A* and *TP53/RB1* was performed in cBioPortal using data sets of four independent studies [23,24,26,27].

To detect alterations in SWI/SNF, PRC1/PRC2 complexes and Ras pathway genes in the bladder cancer cell lines J82 and RT112 as well as bladder cancer tissues, whole exome sequencing was performed. Libraries were constructed from 2 μg non-degraded gDNA (0.7% agarose gel) with SureSelect Human All Exon V6 (probe-covered regions: 60 Mb; Agilent Technologies). Sequencing was carried out on a HiSeq2000 (Ilumina) at the Cologne Center for Genomics. Data analysis was performed using the VARBANK pipeline v.2.3 and the corresponding filter interface (unpublished, https://varbank.ccg.uni-koeln.de/). Illumina Realtime Analysis (RTA) software v.1.8 was applied to filter primary data according to signal purity. Sequencing reads were subsequently aligned against the human genome reference build hg19 using the BWA alignment algorithm [28]. Duplicated reads were marked, reads at known InDel sites realigned and base quality scores corrected using the GATK v1.6 software [29].

Absence of genetic alterations in SWI/SNF and PRC2 subunit genes in the cell line SCaBER was verified using sequencing data provided by the COSMIC database (https://cancer.sanger.ac.uk/cell_lines). Described *ARID1A* truncating mutations in the bladder cancer cell lines HT1376, JMSU-1 and VM-CUB-1 [30] have been verified by Sanger sequencing (S1 Fig).

### Kaplan-Meier survival analysis

Overall survival (OS) of bladder cancer patients showing *ARID1A* gene alterations assumed to result in loss of *ARID1A* gene function (truncating mutations and homozygous deletions) was compared to OS of patients exhibiting putative passenger mutations, gene amplification/gain and wildtype *ARID1A* gene sequence using publicly available sequencing data of two TCGA cohorts comprising 127 [24] and 408 [23] muscle-invasive bladder cancer cases. Clinical follow-up and data on *ARID1A*’s mutational status of the above mentioned studies can be accessed and downloaded via the cBioPortal (http://www.cbioportal.org/) [25].

### Clinical specimens

Formalin-fixed, paraffin-embedded (FFPE) tumorous and normal urothelial tissue samples used to construct a tissue microarray (TMA) were obtained from the diagnostic archive of the Institute of Pathology, Medical Faculty of the RWTH Aachen. This retrospective, anonymized study was conducted in accordance with local Institutional Review Board (IRB)-approved protocols (approval no. EK291/16) of the Medical Faculty at RWTH Aachen University and the principles expressed in the Declaration of Helsinki. The TMA comprised 362 tumor and 21 normal urothelial samples from 284 patients. Clinico-pathologic variables of urothelial bladder cancer cases included in the tissue microarray are summarized in Table 1.

**Table 1:** Clinico-pathological data of all tumor cases analyzed by immunohistochemistry in this study.

**Patients (n = 284)**					
Age	median	69 years (range 27–93)			
Sex	male	206			
	female	78			
**Tumor Cases (n = 362)**		**G3**	**G2 (high-grade)**	**G2 (low-grade)**	**G1**
pTis	175	175	0	0	0
pTa	92	27	3	15	47
pT1	31	31	0	0	0
pT2	33	30	3	0	0
pT3	20	18	2	0	0
pT4	11	11	0	0	0

### Immunohistochemistry

For pre-treatment, TMA sections (2 μm) were incubated in antigen retrieval solution with pH 6 or 9 (PT Link, Dako) at 95°C for deparaffinization, rehydration and epitope retrieval. FFPE slides were then treated with EnVision^TM^ Flex peroxidase blocking reagent (Dako) for 5 min to block endogenous peroxidase activity. Immunostaining was performed with antibodies specific for ARID1A (1:250, D2A8U, Cell Signaling), EZH2 (1:50, 6A10, Leica) and H3K27me3 (1:500, C36B11, Cell Signaling). Subsequently, tissue sections were incubated with a HRP-conjugated secondary reagent (Dako) for 15 min. The peroxidase reaction was visualized with the DAB+ Substrate Chromogen System (Dako). The sections were then counterstained using Mayer`s haematoxylin. Nuclear expression of ARID1A, EZH2 and H3K27me3 was assessed by two experienced pathologists (RK and NTG) according to an adapted immunoreactive score (IRS) developed by Remmele and Stegner (1987) [31].

### Immunocytochemistry

To analyze the subcellular ARID1A protein expression in J82 cell clones, cell pellets were fixed in 4% formalin solution for 30 min at room temperature. Subsequently, fixed cells were embedded in 3% agarose followed by a dehydratization step. Dehydrated agarose-embedded cells were finally covered with paraffin. After preparation of FFPE sections on microscopic slides, ARID1A protein detection was accomplished following the protocol for immunohistochemistry described above.

### Western blotting

Total cell protein lysates were obtained by sonification of cells in an appropriate volume of *1×NuPAGE LDS Sample Buffer* (Invitrogen) supplemented with 50mM dithiothreitol (Life Technologies). Heat denatured samples were loaded on 4–12% gradient gels (NuPAGE; Invitrogen) and then transferred onto 0.2μm PVDF membranes (Whatman) (1h, 100V) for immunodetection. Blots were blocked in TRIS-buffered saline (TBS) containing 0.1% Tween-20 (TBS-T) and either 5% non-fat dry milk (Merck) or BSA (Roth) for 1h at room temperature. Blocked blots were then incubated with the primary antibody overnight at 4°C, diluted in blocking solution either containing 5% non-fat dry milk or BSA. The following primary antibodies were used: ACTB (1:1000, A5441, Sigma-Aldrich), ARID1A (1:1000, D2A8U), CCND1 (1:1000, 92G2), EZH2 (1:1000, D2C9), H3K27me3 (1:1000, C36B11), MYC (1:1000, D84C12), p21 (1:1000, 12D1) (all Cell Signaling), p16 (1:200, MX007, Immunologic), p53 (1:500, D0-1, Santa Cruz). After washing three times (TBS + 0.1% Tween-20), blots were incubated with secondary peroxidase-conjugated antibodies (DAKO) diluted in blocking solution containing 5% non-fat dry milk for 1h at room temperature. After washing three times (TBS + 0.1% Tween-20), antibody detection was accomplished with Pierce ECL Western blotting Substrate (Thermo Scientific).

### RNA extraction and reverse transcription PCR

Total RNA from human cell lines was isolated using the standard procedure for *TRIzol®* (Invitrogen) RNA extraction. Extracted RNA was quantified using the *NanoDrop ND1000* spectrophotometer (Thermo Scientific). The A260 nm/A280 nm ratio was generally between 1.9 and 2.0. Subsequently, cDNA was synthesized using 1μg of total RNA and the reverse transcription system (Promega) as described previously [32]. After cDNA synthesis, enzyme was heat inactivated by incubation for 5min at 95°C. cDNA was stored at -20°C until use.

### Semi-quantitative real-time PCR

cDNAs were amplified by semi-quantitative real-time PCR using *SYBR Green PCR mix* (Bio-Rad Laboratories) and the iCycler *IQ5* (Bio-Rad Laboratories) as described previously [32]. Gene-specific oligonucleotides were designed using *Primer3web* software (version 4.0.0) (http://primer3.ut.ee/). All reactions were performed in triplicates including negative controls without cDNA. Specificity of amplicons was confirmed by size estimation on agarose gels and melt curve analysis. Obtained data were analyzed using the comparative Ct (threshold cycle) method. Complete reaction conditions, primer sequences and lengths of amplicons are listed in S1 Table.

### Cell lines

The human urothelial bladder cancer cell lines (J82, JMSU-1, HT1376, VM-CUB-1, RT112), the squamous cell carcinoma bladder cell line SCaBER and the SV40 large T-antigen-immortalized normal urothelial cell line UROtsa were authenticated using Multiplex Cell Authentication by Multiplexion (Heidelberg) as described recently [33]. J82, JMSU-1, RT112, UROtsa cells were cultured in RPMI medium (Life Technologies), whereas VM-CUB-1 and SCaBER cells in DMEM medium (Life Technologies), both supplemented with 10% fetal calf serum (FCS), 2mM L-glutamine, 50U/l penicillin and 50mg/l streptomycin. For cultivation of HT1376 cells, MEM medium (Life Technologies) supplemented with sodium pyruvate (1mM), 10% fetal calf serum (FCS), 2mM L-glutamine, 50U/l penicillin and 50mg/l streptomycin was used. TERT-immortalized normal human urothelial cells (TERT-NHUC) were cultured using the Keratinocyte Growth Medium 2 Kit (Promocell) as described previously [34]. All cell lines were regularly tested for mycoplasma infection using the PCR-based *Venor® GeM Mycoplasma Detection Kit* (Minerva Biolabs).

### RNA interference

To transiently knockdown ARID1A and EZH2 expression, urothelial cells (1-2x10^5^/well) were seeded in six-well plates and cultured overnight. After 24h, culture medium was replaced and cells transfected for 72h using HiPerFect transfection reagent (Qiagen), *ARID1A*-specific siRNAs (20nM) (Qiagen) and *EZH2*-specific siRNAs (40nM) (Qiagen), respectively. Hs_ARID1A_6: 5‘-CTCGGTATCACCGTTGATGAA-3‘; Hs_ARID1A_4: 5‘-CAGAGTTTACTCTGTACGAAT-3‘. Hs_EZH2_6: 5’-CAGGATGGTACTTTCATTGAA-3’; Hs_EZH2_7: 5’-AACCATGTTTACAACTATCAA-3’. Equal amounts of a scrambled siRNA served as a negative control (AllStars Negative Control siRNA, Qiagen). To generate stably transfected ARID1A knockdown single-cell clones, J82 cells were transfected using FuGene HD Transfection Reagent (Promega) following the manufacturer's instructions and the vector pGeneClip Neomycin either encoding an *ARID1A*-specific shRNA (shRNA 1: 5‘-TGATGGAAGTGACTCCACATT-3‘; shRNA 2: 5‘-GGAGCTATCTCAAGATTCATT-3‘) or a scrambled shRNA sequence (5‘-GGAATCTCATTCGATGCATAC-3‘) as negative control (all vectors from Qiagen). Selection of stable single-cell clones was achieved by culturing transfectants in complete growth medium containing 700μg/ml G418 (Life Technologies) for at least two weeks.

### Cell growth assays

Increase in cell number was recorded for UROtsa cells over a period of 96 h. Briefly, cells were seeded in 12-well culture plates (2x10^4^ cells/well) and cultivated for 96 h (20% O_2_, 5%CO_2_, 37°C). Thereafter, the cell number was determined using the CASY® Cell Counter and Analyzer (OLS OMNI Life Science). Colony growth was analyzed by conducting a 2D colony formation assay. Briefly, UROtsa or J82 cells were seeded in six-well plates (1000 cells/well) containing growth medium. Medium was replaced every two days and cells were cultivated for two weeks (20% O_2_, 5% CO_2_, 37°C). Afterwards, cell colonies were fixed and stained using a 0.5% crystal violet staining solution (80% methanol, 10% formaldehyde, 10% ddH_2_O). Densitometrical evaluation of photographs was accomplished by using ImageJ Software (1.45, National Institute of Health, USA). Experiments were performed in triplicate.

### Drug assays

For short-term drug exposure, 1000–3000 cells/per well were seeded in 96-well plates. 24h after cell seeding, GSK126 (Selleckchem) or DMSO as negative control were added to the cells and these were continuously exposed for 72h. Cell viability was finally estimated using CellTiter-Glo® luminescent cell viabilty assay (Promega). For long-term clonogenic assays, cells were seeded (1000 cells/well) in six-well plates and 24h after cell seeding continuously exposed to GSK126 or DMSO control for 10 days. Medium containing fresh GSK126 was replaced every 2–3 days. Cell colonies were fixed and stained using a 0.5% crystal violet staining solution (80% methanol, 10% formaldehyde, 10% ddH_2_O). Densitometrical evaluation of photographs was accomplished by using ImageJ Software (1.45, National Institute of Health, USA). Experiments were performed in triplicate. Growth IC50 values were determined by plotting lines of best fit using a non-linear regression model. All experiments were performed in triplicate.

### Statistical analysis

For statistical analyses SPSS 19.0 (SPSS, Chicago, IL, USA) and GraphPad Prism 5.0 (Graph-Pad Software, La Jolla, CA, USA) were applied. Differences were considered significant if the two-sided P values were < 0.05. To determine the potential prognostic value of *ARID1A* genetic alterations, univariate Kaplan-Meier survival analysis was performed. Statistical significance was assessed using the univariate log-rank test. For comparison of two groups, the unpaired t test and the Mann-Whitney-U test were applied where appropriate, whereas the Kruskal-Wallis test (Dunn’s multiple comparison post test) and the repeated measures ANOVA (Tukey’s multiple comparison test) test were used to compare more than two groups.

## Results

### *ARID1A* is the most commonly altered subunit gene of SWI/SNF complexes in human urothelial bladder cancer

**Fig 1:**
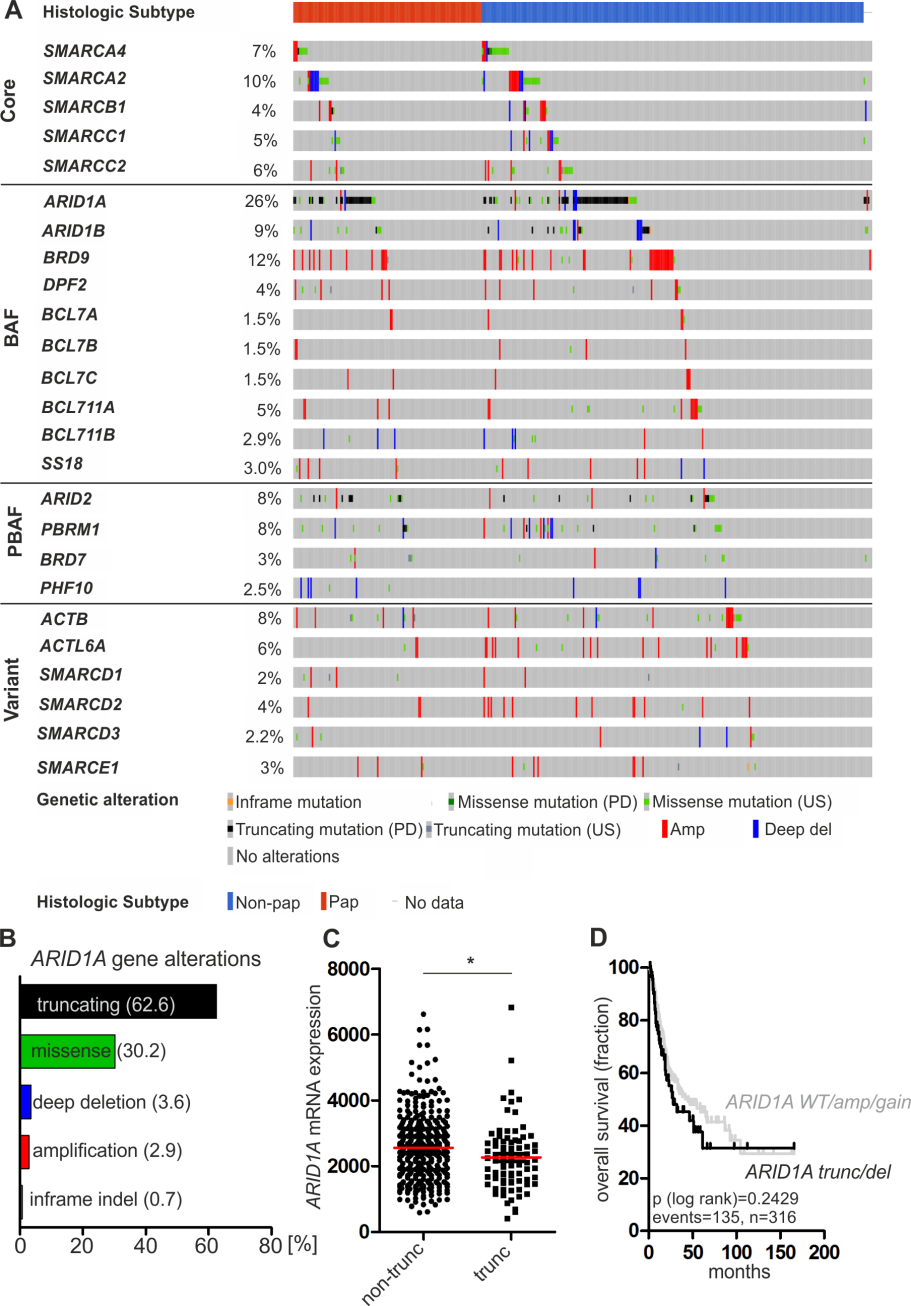
*ARID1A* is the most commonly altered subunit gene of SWI/SNF complexes in human urothelial bladder cancer. (**A**) SWI/SNF subunit gene alterations including *ARID1A* are present in 290 cases (71%) of a TCGA cohort comprising 408 muscle-invasive urothelial bladder carcinomas with 106 patients (26%) showing *ARID1A* gene alterations. The 25 depicted subunit genes are categorized into „core“, „BAF-specific (BAF)“, „PBAF-specific (PBAF)”and „variant/accessory (variant)”subunit genes. Note: nBAF (neuronal BAF)-specific subunit genes have been excluded. US: unknown significance, PD: putative driver, Amp: amplification, Deep del: deep deletion, Non-pap: non-papillary, pap: papillary. (**B**) Frequencies of *ARID1A* gene alteration types in the TCGA cohort. (**C**) *ARID1A* mRNA expression levels in patient samples harboring *ARID1A* truncating mutations (trunc, n = 81) compared to specimens without these alterations (non-trunc, n = 327). * P < 0.05 (unpaired t test). Horizotal line (red): median expression. (**D**) Univariate Kaplan-Meier survival curves displaying overall survival of patients harboring *ARID1A* truncating mutations (trunc) and deep deletions (del) (n = 83; black curve) in relation to patients with *ARID1A* gene amplification (amp), gene gain (gain) and wildtype (WT) *ARID1A* gene sequence (n = 233; grey curve). Cases with *ARID1A* missense mutations of unknown significance and shallow *ARID1A* gene deletions have been excluded from the analysis.

To comprehensively dissect the frequency of inactivating alterations in SWI/SNF complexes in human urothelial bladder carcinomas, we performed a systematic analysis of genomic alterations (mutations and CNVs) in 25 known SWI/SNF subunit genes using a publicly available bladder cancer cohort comprising 412 muscle-invasive bladder cancer (MIBC) cases [23]. For 408 samples sequencing and CNV data were available. The BAF-specific subunit gene *ARID1A* was identified as the by far most commonly altered subunit of SWI/SNF complexes in urothelial carcinomas (106/408 = 26%) with inactivating truncating mutations (frameshift and splice mutations resulting in premature stop codons as well as nonsense mutations) being the predominant mutational type (63% of *ARID1A* gene alterations) followed by alterations in the genes *BRD9* (12%) and *SMARCA2* (10%) (Fig 1A and 1B). Interestingly, *ARID1A* alterations are present in both subgroups of MIBC, i.e. in cases potentially arising from high-grade papillary carcinomas (pTa HG) (35/133 = 26%) and tumors of non-papillary origin developing from carcinoma *in situ* (67/269 = 25%) (Fig 1A). Similar observations have been made by analyzing the previous TCGA cohort from 2014 (S2 Fig).

Subsequently, we analyzed if *ARID1A* truncating mutations are correlated with reduced *ARID1A* mRNA expression as expected from prelimary work [35]. Indeed, we observed a significant (P < 0.05) reduction of *ARID1A* mRNA levels in cases exhibiting truncating mutations compared to patient samples without these alterations (missense mutations, inframe indels and wildtype sequence) (Fig 1C). This observation also remained stable after exclusion of cases showing copy number variations of the *ARID1A* gene locus (not shown).

We next asked if *ARID1A* gene alterations potentially resulting in loss of functional ARID1A protein (truncating mutations and deep deletions) are associated with worse patient prognosis. Comparing overall survival (OS) of patients harboring truncating mutations and deep deletions (median OS: 28.2 months) to those patients showing *ARID1A* gene amplification/gain and wildtype gene sequence (median OS: 44.9 months) revealed no significant association of potentially deleterious *ARID1A* genetic alterations with overall survival (Fig 1D). Comparable results were obtained by analyzing the TCGA 2014 cohort (S2 Fig).

### ARID1A protein expression loss in high-grade urothelial bladder cancer including carcinoma *in situ*

Our comprehensive SWI/SNF subunit gene analysis revealed *ARID1A* truncating mutations, shown to be associated with reduction in *ARID1A* mRNA expression levels (see Fig 1C) and ARID1A protein loss in previous studies [35,36], as the most common genetic alterations of SWI/SNF complexes in urothelial bladder cancer. Current ARID1A protein expression data in urothelial bladder cancer are contradictory, incomplete and conclusions drawn are limited due to small cohort sizes [37–40]. We therefore aimed to perform a thorough, comprehensive IHC-based ARID1A protein expression analysis in a large cohort of urothelial bladder carcinomas including carcinoma in situ (CIS) cases.

**Fig 2:**
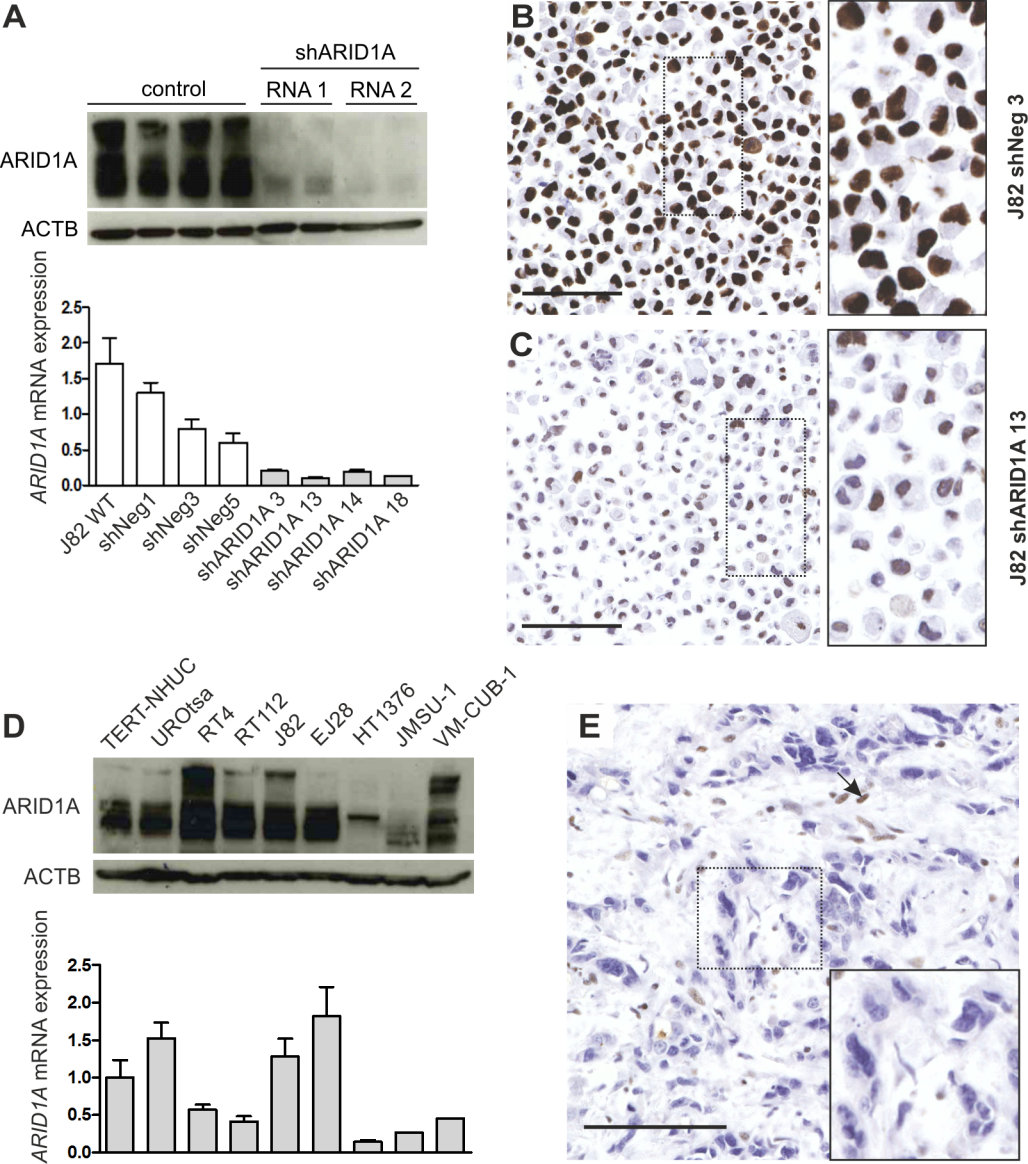
*ARID1A* truncating mutations are associated with reduction in ARID1A protein expression. **(A)** ARID1A expression in J82 wildtype (WT) cells and J82 single-cell clones, either transfected with an *ARID1A*-specific shRNA or a scrambled shRNA control, on protein (upper panel) and mRNA level (bottom panel). ACTB served as a loading control for western blot analysis. *ARID1A* mRNA expression levels are normalized to median mRNA expression of J82 control cells. (**B**-**C**) ARID1A protein detection in fixed, hematoxylin-stained J82 cells transfected with a vector expressing a scrambled shRNA (J82 shNeg 3) (**B**) and an *ARID1A*-specific shRNA sequence (J82 shARID1A 13) (**C**). Bar: 100μm. (**D**) ARID1A expression in urothelial bladder cell lines on protein (upper panel) and mRNA level (bottom panel). HT1376 and JMSU-1 cells, both harboring a homozygous *ARID1A* frameshift mutation (HT1376: p.S186fs*209, JMSU-1: p.R911fs) show strongly reduced ARID1A mRNA and protein expression. ACTB served as a loading control for western blot analysis. *ARID1A* mRNA levels are expressed relative to *ARID1A* mRNA expression in TERT-NHUC cells. (**E**) Muscle-invasive urothelial bladder carcinoma exhibiting an *ARID1A* frameshift deletion (p.P1314Lfs*14) lacks ARID1A protein expression in cancer cells. Arrow: stromal cells expressing ARID1A protein. Bar: 100μm.

We started our analysis with a profound specificity validation of the ARID1A antibody used. First, two vectors, each encoding a different *ARID1A*-specific shRNA (shRNA1 and 2), were applied to knockdown endogenously expressed ARID1A mRNA and protein in J82 cells. As a control, J82 wildtype cells were transfected using the same vector backbone containing a scrambled shRNA sequence (Fig 2A). As expected, nuclear ARID1A protein expression was significantly reduced in single-cell clones transfected with an *ARID1A*-specific shRNA compared to control cells. A representative immunocytochemistry staining is shown in Fig 2B and 2C.

Next, we tested the antibody’s ability to detect urothelial cancer samples containing known truncating *ARID1A* mutations, supposed to show ARID1A protein loss. In line with our assumption, we observed strongly reduced ARID1A mRNA and protein expression in the two bladder cancer cell lines HT1376 and JMSU-1, both harboring a homozygous frameshift mutation in the *ARID1A* gene as validated by Sanger sequencing (Fig 2D and S1 Fig). Of note, no obvious reduction of ARID1A mRNA and protein levels were observed in the cell line VM-CUB-1, displaying a heterozygous *ARID1A* nonsense mutation (Fig 2D and S1 Fig). Moreover, complete absence of ARID1A protein in a human high-grade muscle-invasive urothelial carcinoma, exhibiting a truncating frameshift deletion in the *ARID1A* gene, was noticed (Fig 2E).

**Fig 3:**
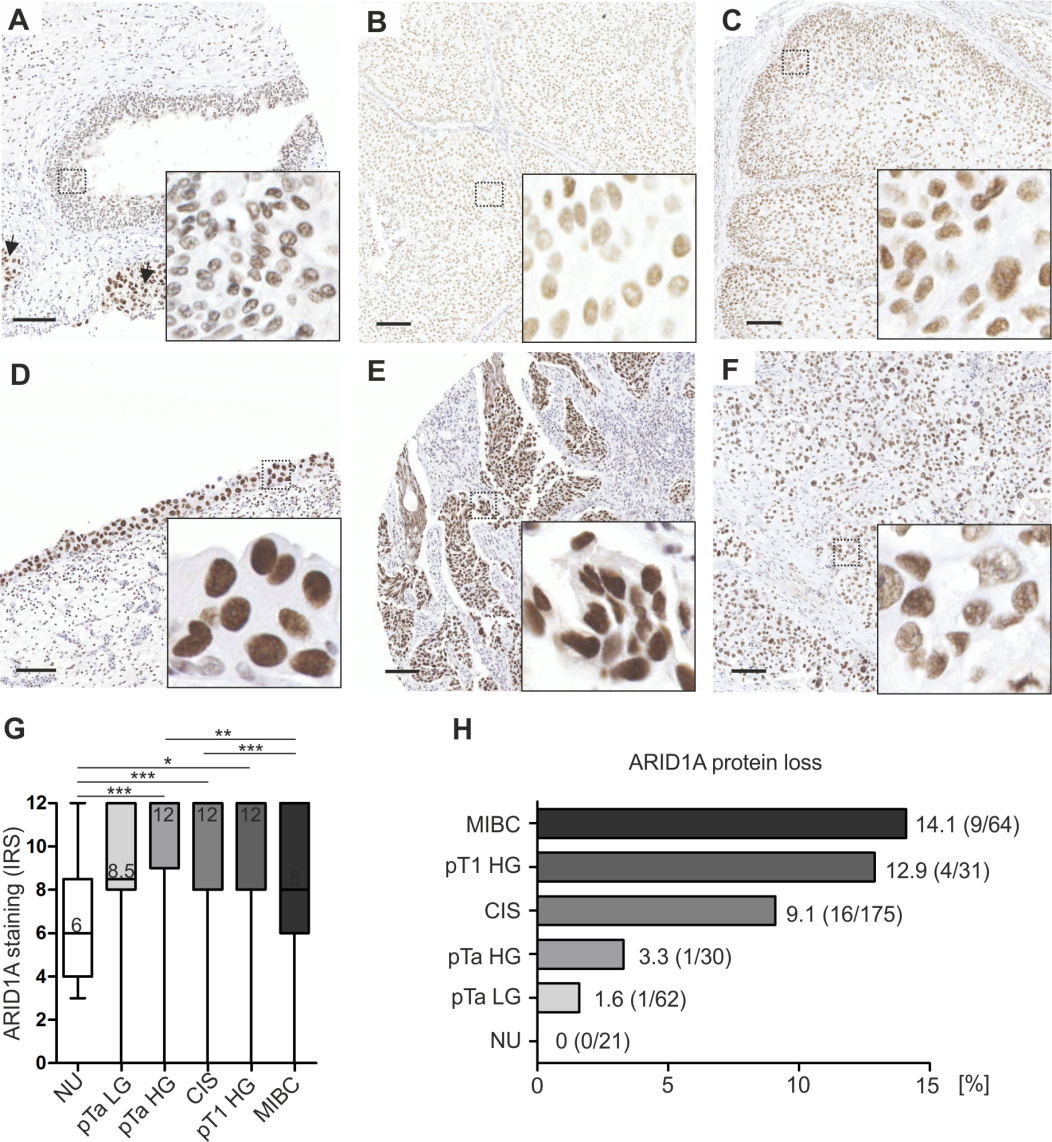
ARID1A protein expression in urothelial carcinomas of different stage and grade compared to normal urothelium of the urinary bladder. (A-F) Immunohistochemical images representative of observed median ARID1A protein expression in normal urothelium of the urinary bladder (NU, n = 21) (A), pTa low-grade (pTa LG, n = 62) (B) and pTa high-grade (pTa HG, n = 30) tumors (C), carcinoma *in situ* (CIS, n = 175) (D), pT1 high-grade (pT1 HG, n = 31) (E) and muscle-invasive bladder cancers (MIBC, n = 64) (F). Arrows in A: CIS. Bar: 100μm. (G) Quantification of ARID1A protein expression by box plot analysis. Horizontal lines/numbers: grouped medians. Boxes: 25–75% quartiles. Vertical lines: range, minimum and maximum. Only significant differences are shown. * P < 0.05, ** P <0.01, *** P < 0.001 (Kruskal-Wallis-test, Dunn’s multiple comparison post test), IRS: immunoreactive score. (H) Quantification of ARID1A protein expression loss (IRS = 0–2).

Subsequently, we applied the validated antibody to analyze a large cohort of urothelial bladder carcinomas (n = 362) and normal urothelial (NU) controls (n = 21) for ARID1A protein expression loss as a potential surrogate for *ARID1A* truncating mutations. For patient characteristics see Table 1. Whereas a moderate ARID1A protein expression was observed in normal urothelial samples (median Remmele Score: 6), ARID1A protein levels were increased in all carcinoma subgroups, with strongest expression in high-grade papillary tumors, namely pTa high-grade (median Remmele score: 12, P < 0.001), CIS (median Remmele score: 12, P < 0.001) and pT1 high-grade cases (median Remmele score: 12, P < 0.05) (Fig 3A–3G). Importantly, while median ARID1A protein expression was increased in all tumor subgroups compared to NU controls, the percentage of cases showing ARID1A protein loss (Remmele score: 0–2) positively correlated with increasing stage and grade (NU = 0, pTa LG = 1.6, pTa HG = 3.3, CIS = 9.1, pT1 HG = 12.9, MIBC = 14.1%) (Fig 3H).

### Analysis of EZH2 inhibition as potential therapeutic strategy for ARID1A-deficient urothelial bladder carcinomas

**Fig 4:**
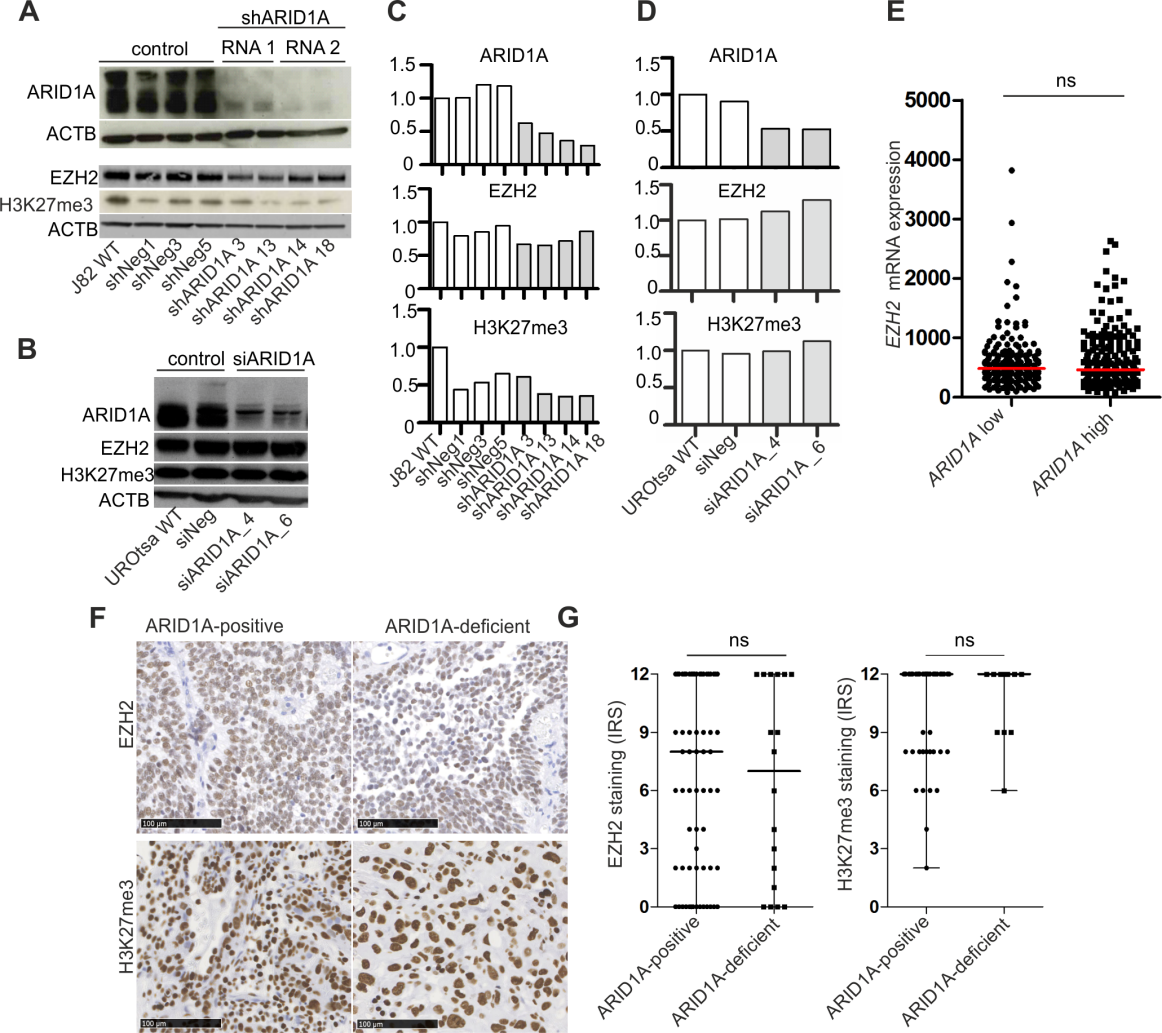
ARID1A-depletion in urothelial cells does neither increase EZH2 protein amount nor H3K27me3 levels. ARID1A, EZH2 protein expression and amount of tri-methylated H3K27 (H3K27me3) in stably transfected J82 single-cell clones (**A**) and transiently transfected UROtsa cells (**B**). ACTB served as a loading control for western blot analysis. WT: wildtype. Densitometrical evaluation of the western blot results shown in A and B are depicted in (**C**) and (**D**), respectively. (**E**) *EZH2* mRNA expression in urothelial carcinomas of the TCGA 2017 data set exhibiting “low” (n = 204) and “high” *ARID1A* (n = 204) mRNA expression. Dichotomization into both groups is based on median *ARID1A* mRNA levels in all tumor samples. ns: not significant (unpaired t test). (**F**) EZH2 protein expression and amount of tri-methylated H3K27 (H3K27me3) in ARID1A-positive (median ARID1A Remmele score: 12, n = 84 for EZH2 cohort and n = 105 for H3K27me3 cohort) as well as ARID1A-deficient (median ARID1A Remmele score: 0, n = 18) urothelial bladder carcinoma cases. Immunohistochemical images are representative of observed median expression of EZH2 and H3K27me3 in the respective group. Bar: 100μm. (**G**) Quantification of EZH2 and H3K27me3 levels by box plot analysis. Horizontal lines: grouped medians. Boxes: 25–75% quartiles. Vertical lines: range, minimum and maximum. IRS: immunoreactive score (Remmele score). ns: not significant (Mann-Whitney-U test).

Having demonstrated ARID1A protein loss in a subgroup of high-grade urothelial bladder tumors including CIS cases, we subsequently aimed to analyze a potential dependency of ARID1A-deficient urothelial cells on EZH2 for the first time. To ensure the same genetic background, we generated two different isogenic *in vitro* models. First, J82 high-grade urothelial bladder cancer cells, neither showing inactivating alterations in subunit genes of the SWI/SNF complex nor genetic alterations in the PRC2/PRC1 complex or Ras pathway genes that might disguise the dependence [22] (S2 Table), were used to generate stable ARID1A-knockdown single-cell clones (Fig 4A and 4C). As a second *in vitro* model we transiently knocked down ARID1A expression in SV40 large T-antigen-immortalized normal urothelial UROtsa cells (Fig 4B and 4D). Regarding both models, ARID1A protein depletion did neither result in up-regulation of EZH2 protein expression nor increased levels of tri-methylated H3K27 (Fig 4A–4D). In accordance with the *in vitro* data, we did not observe differential *EZH2* mRNA amounts comparing bladder carcinomas of the TCGA 2017 data set either showing high *ARID1A* mRNA levels or low *ARID1A* mRNA expression (Fig 4E). Moreover, amounts of EZH2 protein and H3K27me3 were not significantly different, comparing urothelial bladder carcinomas strongly expressing ARID1A protein (median Remmele score: 12) to bladder cancer cases exhibiting ARID1A protein loss or only residual ARID1A expression (median Remmele score: 0) (Fig 4F and 4G).

**Fig 5:**
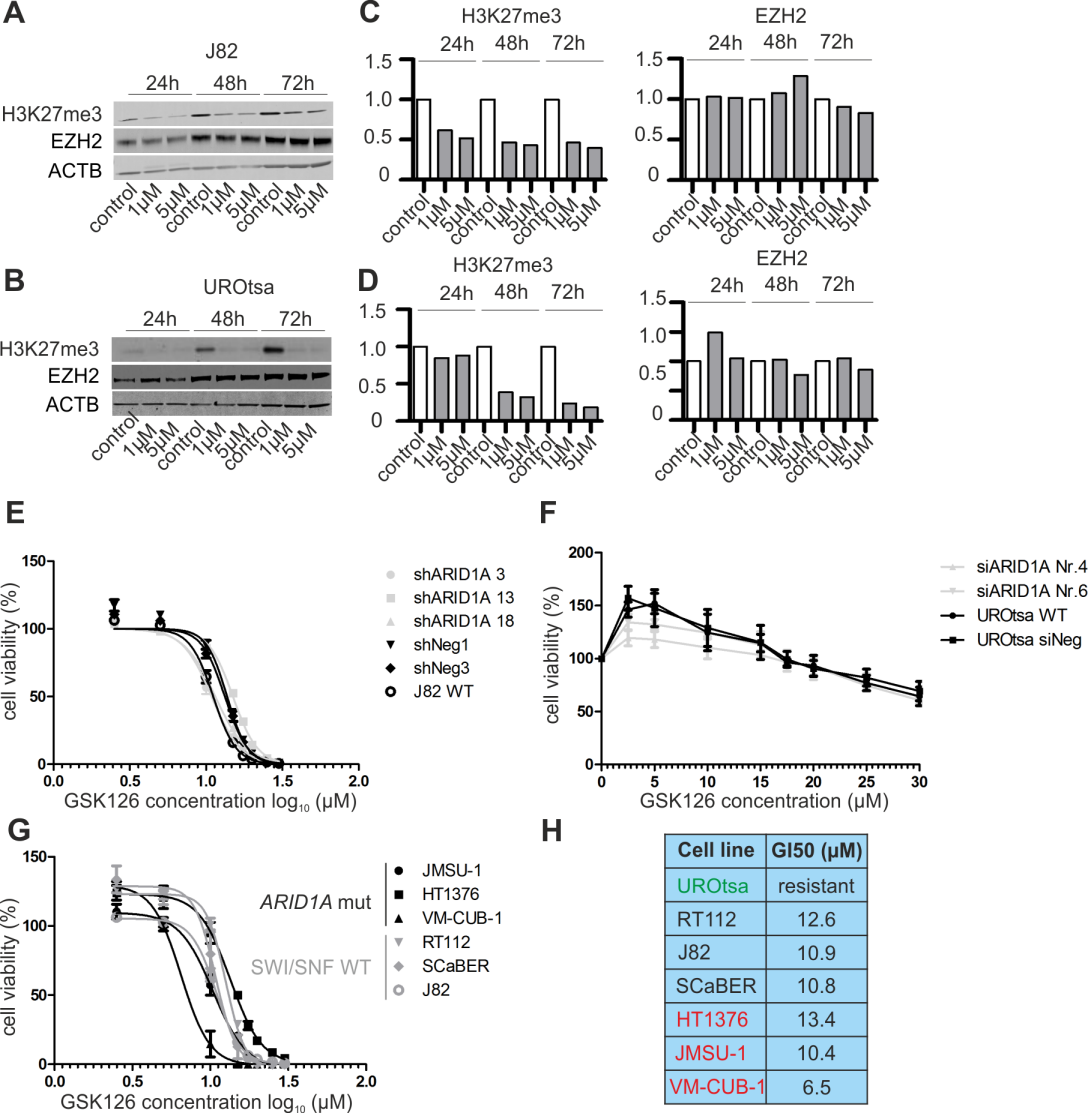
ARID1A-deficient urothelial cells show no enhanced sensitivity towards inhibition of enzymatic EZH2 activity. Amount of tri-methylated H3K27 (H3K27me3) and EZH2 protein expression in J82 (**A**) and UROtsa (**B**) wildtype cells following GSK126 treatment for 24/48/72 hours in the indicated concentrations in comparison to DMSO control. Densitometrical evaluation of the western blot results shown in A and B are depicted in (**C**) and (**D**), respectively. (**E**) Dose-response curves for J82 ARID1A-depleted single-cell clones and controls treated with the indicated GSK126 concentrations for 72h. Error bars (n = 3): SEM. (**F**) Dose-response curves for UROtsa ARID1A-depleted cells and controls treated with the indicated GSK126 concentrations for 72h. Error bars (n = 3): SEM. (**G**) Dose-response curves for bladder cancer cell lines without genetic SWI/SNF alterations (RT112, SCaBER, J82) and *ARID1A*-mutated cells (HT1376, JMSU-1, VM-CUB-1) treated with the indicated GSK126 concentrations for 72h. Error bars (n = 3): SEM. WT: wildtype. (**H**) Determined growth IC50 (GI50) values for all cell lines treated with GSK126 for 72h. Green label: normal urothelial model UROtsa, black label: cell lines wihout genetic SWI/SNF alterations, red label: *ARID1A*-mutated cell lines.

Subsequently, ARID1A-depleted and control cells of both *in vitro* models were treated with different concentrations of the small-molecule EZH2-inhibitor GSK126 [41], that blocks the histone methyltransferase activity of EZH2 leading to decreased H3K27me3 levels without affecting EZH2 protein amount (Fig 5A–5D). However, we did neither observe sensitization of ARID1A-depleted J82 nor UROtsa cells towards GSK126 treatment. In short-term (72 hours drug exposure) cellular viability assays, growth IC50 values for J82 control cells ranged from 11.2–13.7μM compared to 10.9–15.0μM for ARID1A-depleted J82 single-cell clones (Fig 5E). Concerning the UROtsa *in vitro* model, growth IC50 values could not be determined as both controls and ARID1A-depleted cells showed resistance to GSK126 treatment (Fig 5F). In concordance with the results of short-term drug exposure, long-term (10 days GSK126 treatment) clonogenic assays also revealed no differential response to GSK126 treatment (S3 Fig).

Next, we selected three urothelial bladder cancer cell lines harboring *ARID1A* truncating mutations, namely HT1376, JMSU-1, VM-CUB-1 (S1 Fig) and compared their GSK126 treatment response to three bladder cancer cell lines (J82, RT112, SCaBER) without SWI/SNF complex gene alterations. To check for absence of SWI/SNF and PRC2 alterations in J82 and RT112, prorietary whole exome sequencing data were used, whereas absence of these alterations in SCaBER was verified using available sequencing data provided by the COSMIC database. In accordance with our prior observations, presence of *ARID1A* truncating mutations did not predict consistently enhanced GSK126 sensitivity (Fig 5G and 5H): the mean GI50 values were 11.4μM (range: 10.8–12.6μM) and 10.1μM (range: 6.5–13.4μM) for control and *ARID1A*-mutated cells, respectively. Of note, even though highest GSK126 sensitivity was observed for the heterogeneously *ARID1A*-mutated cell line VM-CUB-1, showing relatively high ARID1A protein levels, lowest sensitivity was determined for HT1376, exhibiting a homozygous *ARID1A* truncating mutation and relatively low ARID1A protein levels (see Fig 2D).

Recently, Kim and colleagues suggested that SWI/SNF-deficient cells might be dependent on a non-catalytic function of EZH2 in stabilizing the PRC2 complex [22]. Following this assumption, we transiently silenced EZH2 expression in two stable J82 single-cell clones, either showing *ARID1A* knockdown or *ARID1A* wildtype expression (S4 Fig). Importantly, EZH2 depletion did neither inhibit cellular growth of ARID1A-deficient cells, nor J82 cells with wildytpe ARID1A expression but even enhanced it.

Taken together, our preliminary *in vitro* data do neither support the concept of an anti-regulation between ARID1A and EZH2 in urothelial cells nor ARID1A-deficiency as a suitable predictive biomarker for EZH2-inhibitor treatment response in urothelial bladder cancer.

### ARID1A protein loss in normal urothelial cells affects p53/p21 signaling

The results of recent functional studies suggest that SWI/SNF complexes show both, a functional redundancy to RB1 [6] and p53 [10] in cell cycle control, depending on the cell-type under examination. A potential impact of ARID1A on cell-cycle control of bladder urothelial cells has not been investigated so far. We therefore examined the co-occurence of gene alterations in *ARID1A* and *TP53*/*RB1* taking four independent, publicly available bladder cancer sequencing studies [23,24,26,27] into account. Taken together, we were not able to reveal a clear co-occurence/mutual exclusivity pattern for *ARID1A* and *TP53/RB1* genetic alterations in bladder carcinoma samples (S5 Fig and S3 Table).

**Fig 6:**
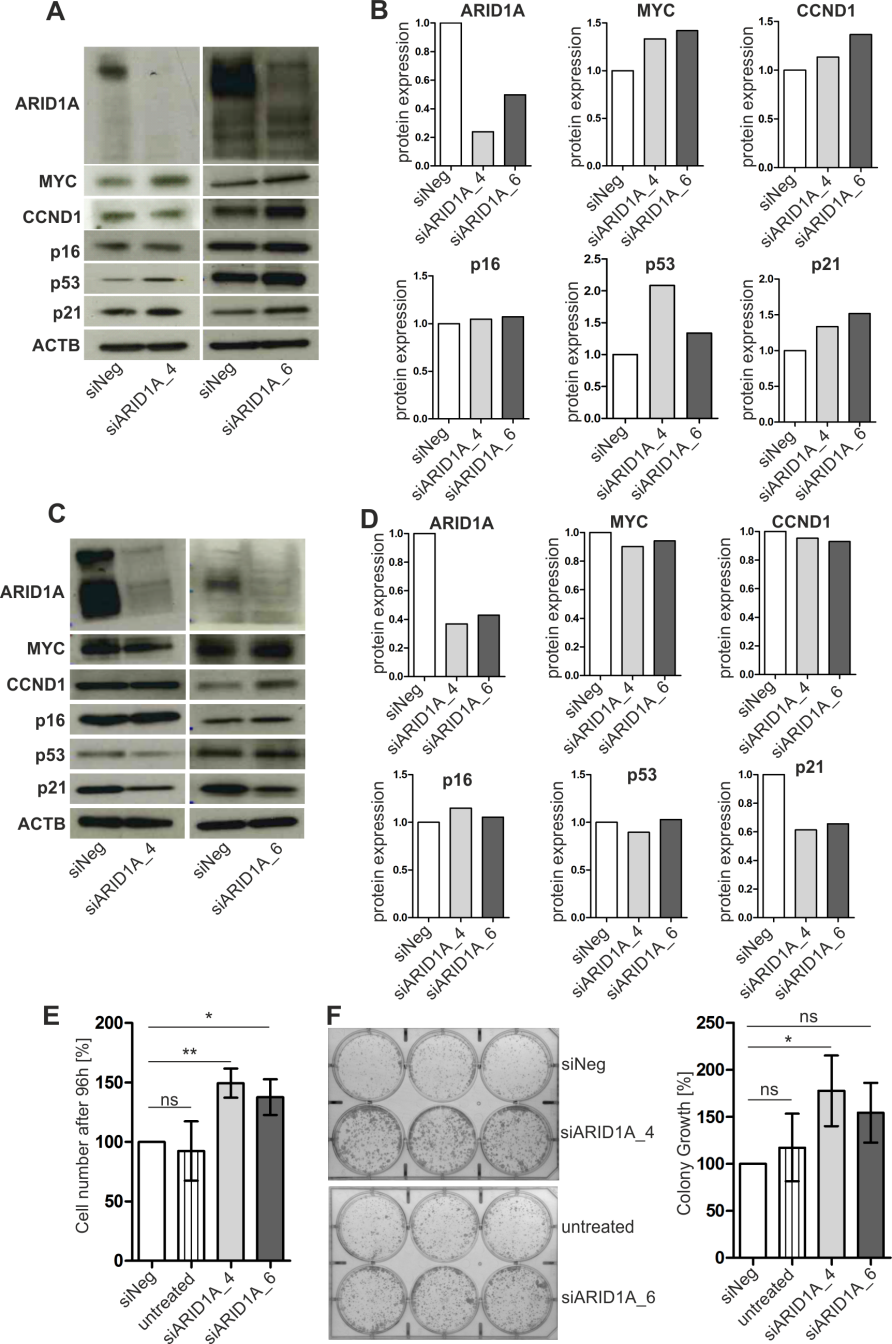
Impact of ARID1A on the expression of cell-cycle-related genes and cell growth of urothelial cells. (**A**) Representative western blot analysis of TERT-immortalized normal human urothelial cells (TERT-NHUC) treated with two different *ARID1A*-specific siRNAs (si*ARID1A_4 and 6*) and a negative control (siNeg). ACTB was used as a loading control. Experiments have been performed in triplicate. (**B**) Densitometrical evaluation of the western blot results shown in A. Protein expression of control cells (siNeg) was set to 1. (**C**) Representative western blot analysis of SV40 large T-antigen-immortalized UROtsa cells treated with two different *ARID1A*-specific siRNAs (si*ARID1A_4 and 6*) and a negative control (siNeg). ACTB was used as a loading control. Experiments have been performed in triplicate. (**D**) Densitometrical evaluation of the western blot results shown in C. Protein expression of control cells (siNeg) was set to 1. (**E**) Cell growth analysis of UROtsa cells treated with two different *ARID1A*-specific siRNAs (si*ARID1A_4 and 6*) in comparison to the siRNA negative control (siNeg) and UROtsa wildtype cells (untreated). The mean cell number after 96 hours cell growth of independent experiments (n = 3) was calculated. The values depicted were normalized to cellular growth of the siNeg control. Vertical lines: standard deviation of triplicates. * P <0.05, ** P <0.01, ns: not significant (repeated measures ANOVA, Tukey’s multiple comparison test). (**F**) Left: representative colony formation assay in six-well plates containing UROtsa cells treated with two different *ARID1A*-specific siRNAs (si*ARID1A_4 and 6*) in comparison to a siRNA negative control (siNeg) and wildtype cells without treatment (untreated) two weeks after cell seeding. Right: Densitometrical evaluation of 2D colony growth of triplicate experiments. The mean colony growth was calculated and the data were normalized to colony growth of the control (siNeg). Horizontal lines: mean values of triplicate experiments. Vertical lines: standard deviation of triplicates. * P <0.05, ns: not significant (repeated measures ANOVA, Tukey’s multiple comparison test).

Subsequently, we performed *in vitro* analyses selecting TERT-immortalized normal human urothelial cells (TERT-NHUC) [34] showing an intact cell cycle as adequate *in vitro* model to analyze potential effects of ARID1A loss on the expression of key cell cycle-related genes (*TP53*, *CDKN1A*, *MYC*, *CDKN2A*, *CCND1*) previously associated with *ARID1A* gene function [14]. Interestingly, ARID1A protein loss resulted in enrichment of wildype p53 protein (range FC: 1.3–2.1) potentially increasing *CDKN1A* transcript (FC: 1.5) and p21 protein (range FC: 1.3–1.5) levels (S6 Fig and Fig 6A and 6B). *MYC* has been described as potential key target gene of ARID1A (SWI/SNF) function [9]. We observed a slight but reproducible increase of MYC mRNA (FC: 1.6) and protein (range FC: 1.3–1.4) levels (S6 Fig and Fig 6A and 6B) in ARID1A-depleted TERT-NHUC in comparison to controls that might in part contribute to cell-cycle deregulation activating p53/p21 signaling.

Due to insufficient *in vitro* growth of TERT-NHUC, we applied a second normal urothelial cell *in vitro* model (UROtsa), that has been immortalized by SV40 large T-antigen, to study the effect of ARID1A on cellular growth [42]. Here, ARID1A knockdown resulted in significant (p < 0.05) stimulation of cell growth and an increased colony forming ability of these cells (Fig 6E and 6F). Analyzing underlying molecular changes in cell cycle-associated genes revealed a striking down-regulation of *CDKN1A* transcript (FC: 3.2) and p21 protein levels (range FC: 1.5–1.6) (S7 Fig and Fig 6C and 6D).

## Discussion

Current bladder cancer therapy mainly relies on non-specific treatments, i.e. chemotherapy or BCG-based immunotherapy in addition to surgery depending on stage and grade of the disease [4,5] highlighting the need for effective, molecular targeted therapies with reduced side effects. SWI/SNF complex subunit genes have been shown to be mutated in ~20% across all human tumor entities with *ARID1A* being the most commonly altered subunit gene [15]. In urothelial bladder cancer, the BAF complex-specific gene *ARID1A* has been identified as one of the top-altered genes with mutational frequencies ranging from 13 to 38% between studies [24,26,27,37,43–47]. Importantly, previous work revealed a functionally antagonistic relationship between SWI/SNF and PRC2 complexes [17], resulting in a proposed dependency of SWI/SNF-mutated carcinomas on EZH2, a histone methyltransferase subunit of the PRC2 complex [21,22]. The aim of the current study was thus to analyze if the suggested synthetic lethality concept is also applicable as a potential therapeutic option for urothelial bladder cancer.

SWI/SNF complexes are assemblies of more than 10 subunits that are encoded by 29 known subunit genes, resulting in a great diversity in SWI/SNF complex composition [13]. Here, we aimed to systematically dissect the frequency of genetic alterations in SWI/SNF subunit genes other than *ARID1A* that might contribute to inactivation of SWI/SNF complexes in urothelial bladder carcinomas. We analyzed mutations and CNVs in 25 SWI/SNF subunit genes (excluding nBAF-specific components from the analysis) making use of publicly available sequencing data for 408 [23] as well as 127 muscle-invasive bladder cancer samples [24] and identified *ARID1A* truncating mutations as the by far most common alterations in SWI/SNF complexes. Although a previously performed meta-analysis considering SWI/SNF subunit gene mutations in several human carcinomas including bladder cancer came to similar results, the conclusions drawn for bladder carcinomas have been limited as sequencing data for only 9 cases have been included [15].

Thus, we subsequently focused on ARID1A analyzing if the frequency of truncating *ARID1A* mutations also translates into a similar frequency of cases showing ARID1A protein loss [35]. Current protein expression data for ARID1A in urothelial bladder cancer are inconsistent and incomplete. Two recent studies observed a reduction of ARID1A protein expression with increasing grade and stage of urothelial carcinomas [37,39] associated with worse patient prognosis. In contrast to that, Faraj et al. reported an up-regulation of ARID1A protein in urothelial bladder tumors compared to NU controls that was related to worse patient survival [38]. Recently, Agaimy and colleagues could not detect loss of ARID1A protein expression in any conventional urothelial bladder cancer case under examination but they observed loss of SWI/SNF components including ARID1A only in rare undifferentiated/dedifferentiated urothelial carcinomas with rhabdoid features [40]. Importantly, the high-grade precursor lesion of most MIBC, the carcinoma *in situ*, has not been considered so far in any of the previously conducted studies. For these reasons we started our analysis with a profound specificity validation of the ARID1A antibody applied and performed a thorough, comprehensive IHC-based ARID1A expression analysis in a large cohort of urothelial bladder carcinomas including carcinoma *in situ* (CIS) cases. We observed increased ARID1A protein levels in all urothelial carcinoma subgroups with strongest expression in early tumor stages compared to NU controls. As the SWI/SNF complex is involved in DNA repair [11,12], this observation is in agreement with recent findings showing activation of the DNA damage response, especially in early tumor stages, as a direct consequence of increased DNA damage [48]. Importantly, the percentage of cases showing ARID1A protein loss was positively correlated with increasing stage and grade of bladder carcinomas that is partially in concordance with recently published work [37,39]. In support of previously published data, showing that *ARID1A* truncating mutations result in ARID1A protein expression loss [35–37], we here provide further evidence for this assumption in urothelial bladder cancer. However, we are aware that also other mechanisms, e.g. gene silencing by promoter hypermethylation [49], might also contribute to *ARID1A* gene expression reduction/loss and should be addressed in future studies.

Previous studies suggested a functional redundancy between SWI/SNF complexes and the RB1 pathway in cell cycle control [6,50]. In contrast to this notion, Guan and colleagues interestingly observed that *ARID1A* and *TP53* mutations are mutually exclusive and that ARID1A acts in concert with p53 to regulate target gene expression including *CDKN1A* in gynecological cancers [10]. Although we did not reveal a clear co-occurence/mutual exclusivity pattern for *ARID1A* and *TP53/RB1* genetic alterations in bladder carcinoma samples using sequencing data of independent studies [23,24,26,27], depletion of ARID1A in TERT-immortalized normal human urothelial cells (TERT-NHUC) resulted in the induction of p53/p21 expression, potentially in part by MYC deregulation [51]. In contrast to ARID1A depletion in the background of an intact cell cycle (TERT-NHUC), ARID1A deficiency in the urothelial model system UROtsa, exhibiting a deregulated cell cycle by SV40 large T-antigen-immortalization, resulted in enhanced cellular growth, probably in part due to downregulation of p21 expression. Interestingly, a recent work has demonstrated a mechanistic link between ARID1A and p53 in the regulation of p21 expression in gynecologic cancers [10]. Even though further investigaton is clearly needed, our preliminary data provide first hints that inactivation of *ARID1A* might not be sufficient to initiate tumor development, but that additional *TP53* inactivation might be necessary to drive oncogenic transformation of urothelial cells. In concordance with this notion, *ARID1A* knockout alone did not result in tumor formation in the murine urinary bladder [7].

Having demonstrated ARID1A protein loss in a significant portion (10%) of high-grade urothelial bladder carcinomas of different stages including CIS, we analyzed for the first time if a functionally antagonistic relationship between ARID1A and EZH2 exists in urothelial cells, and secondly if inhibition of EZH2 methyltransferase activity could be a potential therapeutic option for ARID1A-deficient bladder carcinomas. In contrast to the initial observation that SWI/SNF complex inactivation (by SMARCB1 depletion) results in EZH2 up-regulation and enhanced H3K27-trimethylation in murine embryonic fibroblasts and CD8+ T cells [17], we did neither note an increase in the amount of EZH2 protein nor levels of trimethylated H3K27 in urothelial cells following ARID1A depletion, that is in line with results shown for human ovarian clear cell carcinoma (OCCC) cells [21]. Moreover, we did not observe a correlation of ARID1A expression with EZH2 or H3K27me3 amounts in human bladder carcinomas. Importantly, ARID1A depletion or *ARID1A* truncating mutations did not sensitize urothelial cells to treatment with the small molecule EZH2-inhibitor GSK126, contrasting recent work using several tumor cell lines of different origin (lung, adrenal cortex, ovarian clear cell, endometrial, renal cell and rhabdoid carcinoma cells [21,22]) but excluding urothelial cells from their analyses. Selection of our *ARID1A-*knockdown *in vitro* models (J82 and UROtsa) was based on the absence of inactivating alterations in subunit genes of the SWI/SNF complex as well alterations in PRC2/PRC1 complex and Ras pathway genes [22] that might disguise putative GSK126 sensitization effects based on *ARID1A*-knockdown. Of note, even though further investigation is needed, we observed resistance of the normal urothelial model UROtsa but response of all bladder cancer cells analyzed towards GSK126 treatment (range: 6.5–13.4μM), potentially relevant for clinical translation. The focus of our study was to test the potential of ARID1A-deficiency to predict response to already available EZH2 small-molecule inhibitors such as GSK126 affecting its enzymatic activity [41]. Notably, Kim et al. showed that some SWI/SNF-mutant tumor cell lines are not dependent on EZH2’s methyltransferase activity but rather on a non-catalytic function of EZH2 in stabilizing the PRC2 complex [22]. Therefore, we additionally depleted EZH2 expression in two stable J82 single-cell clones, either showing *ARID1A* knockdown or *ARID1A* wildtype expression. Importantly, EZH2 knockdown did neither inhibit cellular growth of J82 cells deficient for ARID1A nor J82 cells showing wildytpe ARID1A expression. In contrast to current data in bladder cancer [52,53], we even observed an increase in the colony forming ability of EZH2-depleted J82 cells that was more pronounced in the ARID1A-deficient background. Future studies should validate these preliminary observations by including *ARID1A*-mutated and SWI/SNF wildtype bladder cancer cells. Taken together, we assume that ARID1A-deficiency (*ARID1A* truncating mutations and/or expression loss) is not a suitable predictive biomarker for EZH2 inhibitor treatment response in urothelial bladder cancer.

Regarding the high frequency of genetic *ARID1A* alterations in urothelial bladder and a demonstrated loss of ARID1A protein expression, predominantly in a subset of high-grade bladder carcinomas including CIS, it seems worthwile to promote research on the identification of specific vulnerabilities conferred by ARID1A-deficiency. Considering molecular variability between different tumor entities and consequent difficulties in the identification of globally effective cancer treatment strategies, we suggest that bladder cancer-specific high-throughput drug discovery screens should be performed in near future to promote successful discovery of novel targeted drugs.

## Supporting information

S1 Fig Validation of *ARID1A* truncating mutations in bladder cancer cell lines. (PPTX)

S2 Fig SWI/SNF subunit gene alterations in the TCGA 2014 cohort. (DOCX)

S3 Fig Long-term GSK126 treatment of J82 cells. (DOCX)

S4 Fig EZH2 protein depletion in ARID1A-deficient J82 bladder cancer cells. (DOCX)

S5 Fig Genetic alterations in *ARID1A* as well as *TP53* and *RB1*. (DOCX)

S6 Fig mRNA expression of *MYC*, *CDKN1A*, *CCND1* in TERT-NHUC. (DOCX)

S7 Fig mRNA expression of *MYC*, *CDKN1A*, *CCND1* in UROtsa cells. (DOCX)

S1 Table Primer sequences and PCR conditions for RNA expression analyses. (DOC)

S2 Table Genes analyzed in J82 and RT112 cells for genetic alterations. (DOCX)

S3 Table Mutual exclusivity/co-occurrence analysis for *ARID1A*, *TP53*, *RB1*. (DOCX)
