# Supplementary material for: ARID1A-deficiency in urothelial bladder cancer: No predictive biomarker for EZH2-inhibitor treatment response?
Source: PLoS One. 2018 Aug 23;13(8):e0202965. doi: 10.1371/journal.pone.0202965 (PMC6107234; doi:10.1371/journal.pone.0202965)
Supplement: S1 Fig — (PPTX) [file pone.0202965.s001.pptx]

## Slide 1
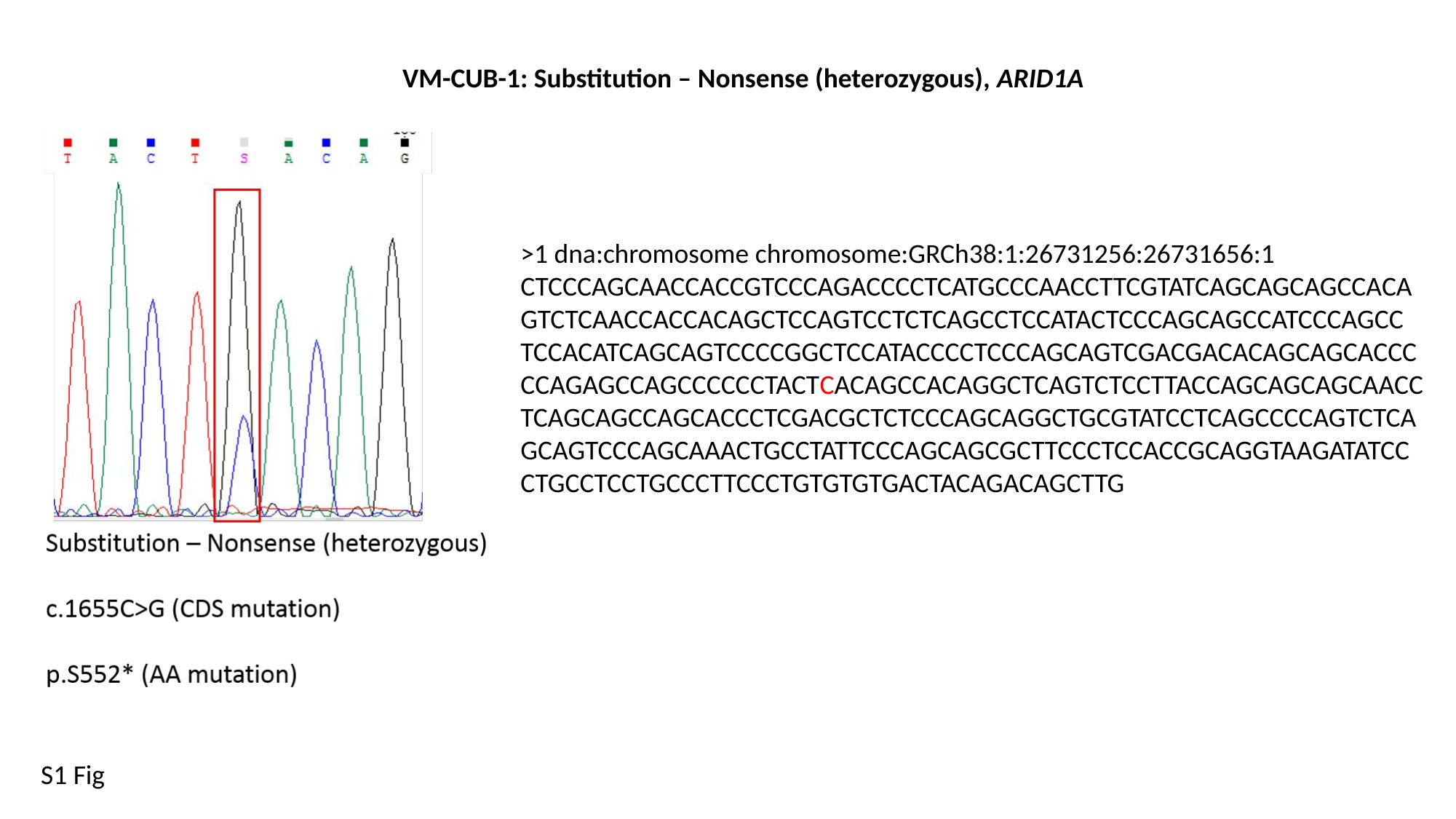

VM-CUB-1: Substitution – Nonsense (heterozygous), ARID1A
>1 dna:chromosome chromosome:GRCh38:1:26731256:26731656:1
CTCCCAGCAACCACCGTCCCAGACCCCTCATGCCCAACCTTCGTATCAGCAGCAGCCACA
GTCTCAACCACCACAGCTCCAGTCCTCTCAGCCTCCATACTCCCAGCAGCCATCCCAGCC
TCCACATCAGCAGTCCCCGGCTCCATACCCCTCCCAGCAGTCGACGACACAGCAGCACCC
CCAGAGCCAGCCCCCCTACTCACAGCCACAGGCTCAGTCTCCTTACCAGCAGCAGCAACC
TCAGCAGCCAGCACCCTCGACGCTCTCCCAGCAGGCTGCGTATCCTCAGCCCCAGTCTCA
GCAGTCCCAGCAAACTGCCTATTCCCAGCAGCGCTTCCCTCCACCGCAGGTAAGATATCC
CTGCCTCCTGCCCTTCCCTGTGTGTGACTACAGACAGCTTG
S1 Fig

## Slide 2
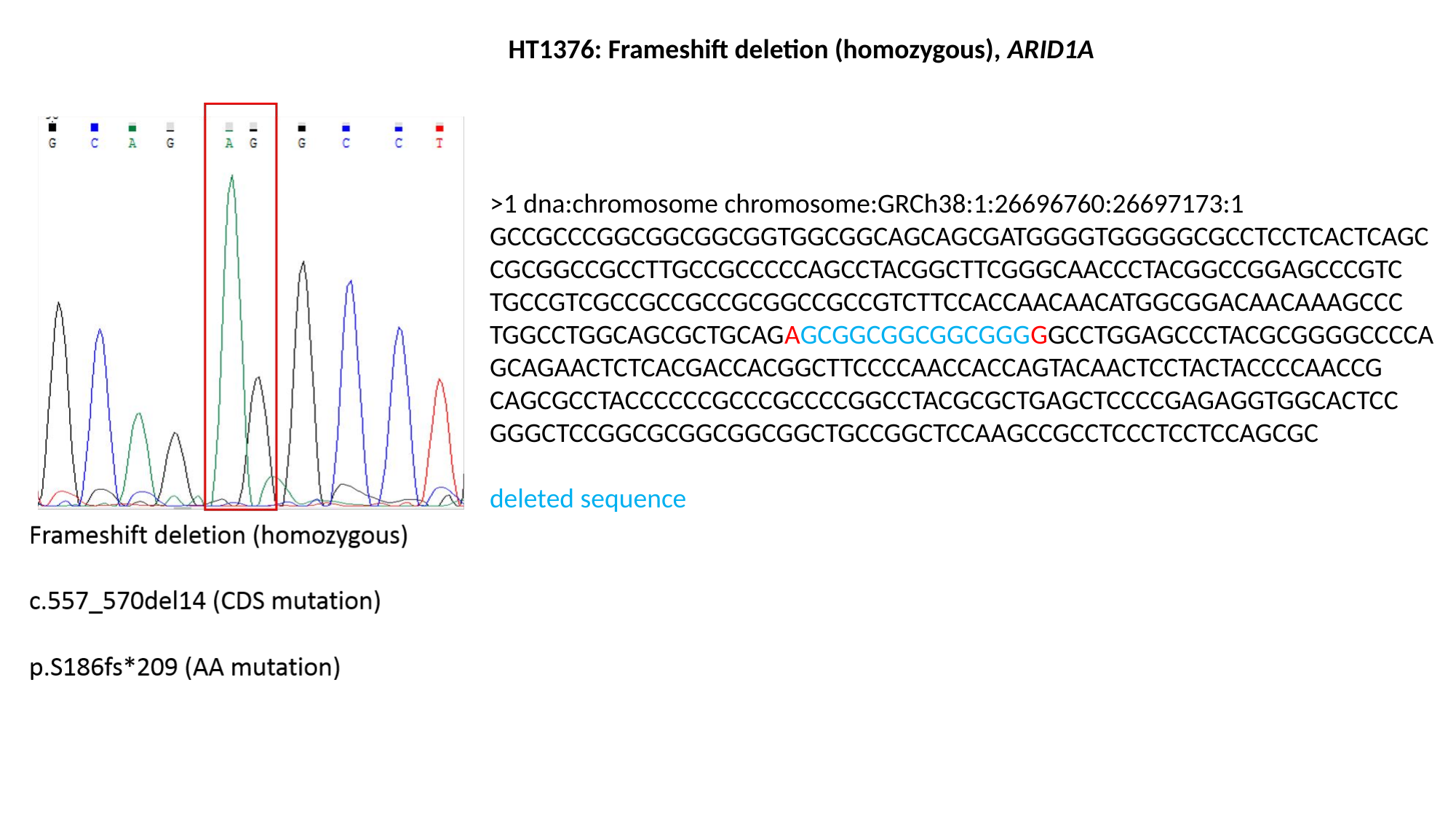

HT1376: Frameshift deletion (homozygous), ARID1A
>1 dna:chromosome chromosome:GRCh38:1:26696760:26697173:1
GCCGCCCGGCGGCGGCGGTGGCGGCAGCAGCGATGGGGTGGGGGCGCCTCCTCACTCAGC
CGCGGCCGCCTTGCCGCCCCCAGCCTACGGCTTCGGGCAACCCTACGGCCGGAGCCCGTC
TGCCGTCGCCGCCGCCGCGGCCGCCGTCTTCCACCAACAACATGGCGGACAACAAAGCCC
TGGCCTGGCAGCGCTGCAGAGCGGCGGCGGCGGGGGCCTGGAGCCCTACGCGGGGCCCCA
GCAGAACTCTCACGACCACGGCTTCCCCAACCACCAGTACAACTCCTACTACCCCAACCG
CAGCGCCTACCCCCCGCCCGCCCCGGCCTACGCGCTGAGCTCCCCGAGAGGTGGCACTCC
GGGCTCCGGCGCGGCGGCGGCTGCCGGCTCCAAGCCGCCTCCCTCCTCCAGCGC
deleted sequence

## Slide 3
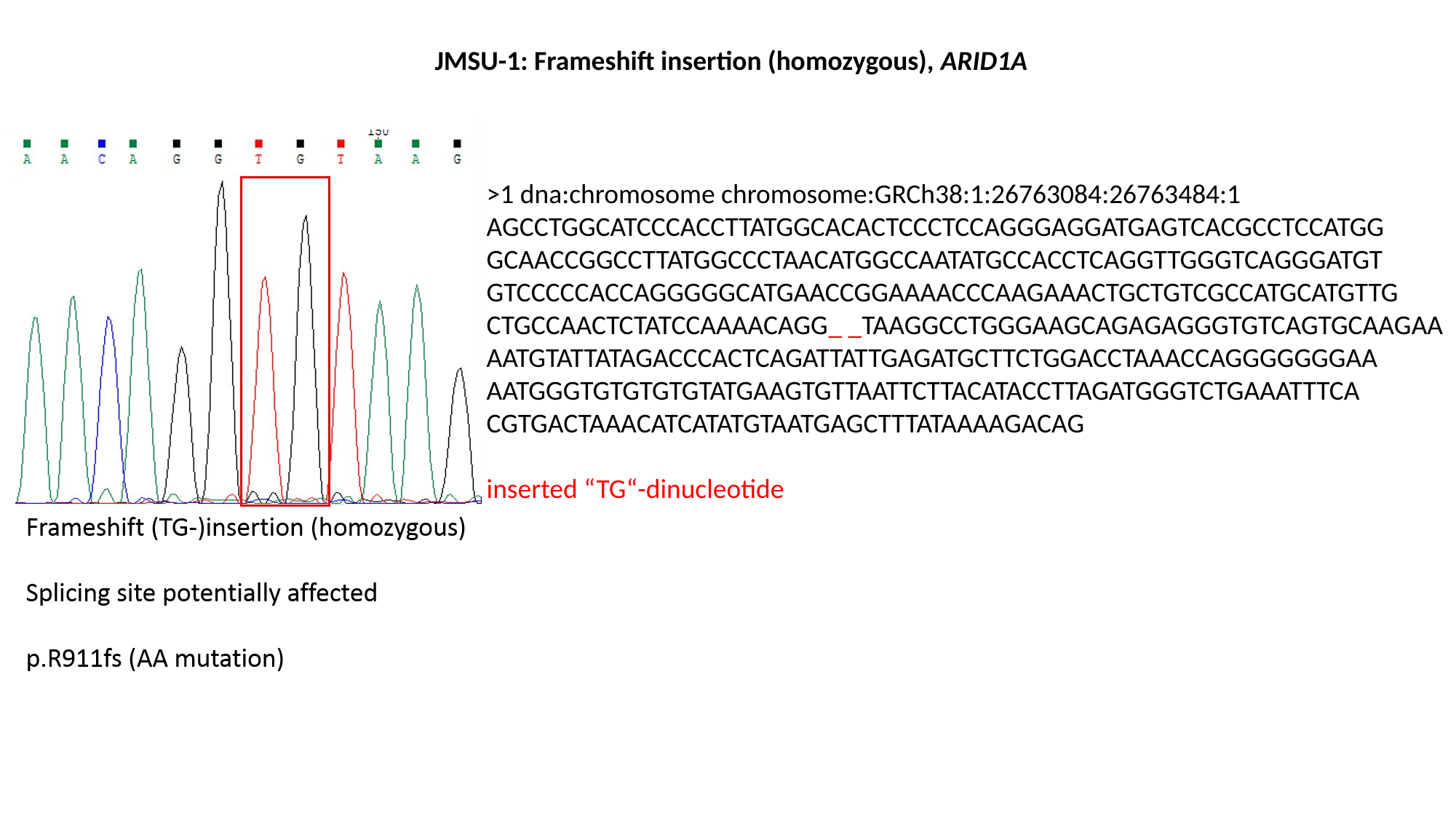

JMSU-1: Frameshift insertion (homozygous), ARID1A
>1 dna:chromosome chromosome:GRCh38:1:26763084:26763484:1
AGCCTGGCATCCCACCTTATGGCACACTCCCTCCAGGGAGGATGAGTCACGCCTCCATGG
GCAACCGGCCTTATGGCCCTAACATGGCCAATATGCCACCTCAGGTTGGGTCAGGGATGT
GTCCCCCACCAGGGGGCATGAACCGGAAAACCCAAGAAACTGCTGTCGCCATGCATGTTG
CTGCCAACTCTATCCAAAACAGG_ _TAAGGCCTGGGAAGCAGAGAGGGTGTCAGTGCAAGAA
AATGTATTATAGACCCACTCAGATTATTGAGATGCTTCTGGACCTAAACCAGGGGGGGAA
AATGGGTGTGTGTGTATGAAGTGTTAATTCTTACATACCTTAGATGGGTCTGAAATTTCA
CGTGACTAAACATCATATGTAATGAGCTTTATAAAAGACAG
inserted “TG“-dinucleotide
