# Supplementary material for: ARID1A-deficiency in urothelial bladder cancer: No predictive biomarker for EZH2-inhibitor treatment response?
Source: PLoS One. 2018 Aug 23;13(8):e0202965. doi: 10.1371/journal.pone.0202965 (PMC6107234; doi:10.1371/journal.pone.0202965)
Supplement: S2 Fig — (DOCX) [file pone.0202965.s002.docx]

**
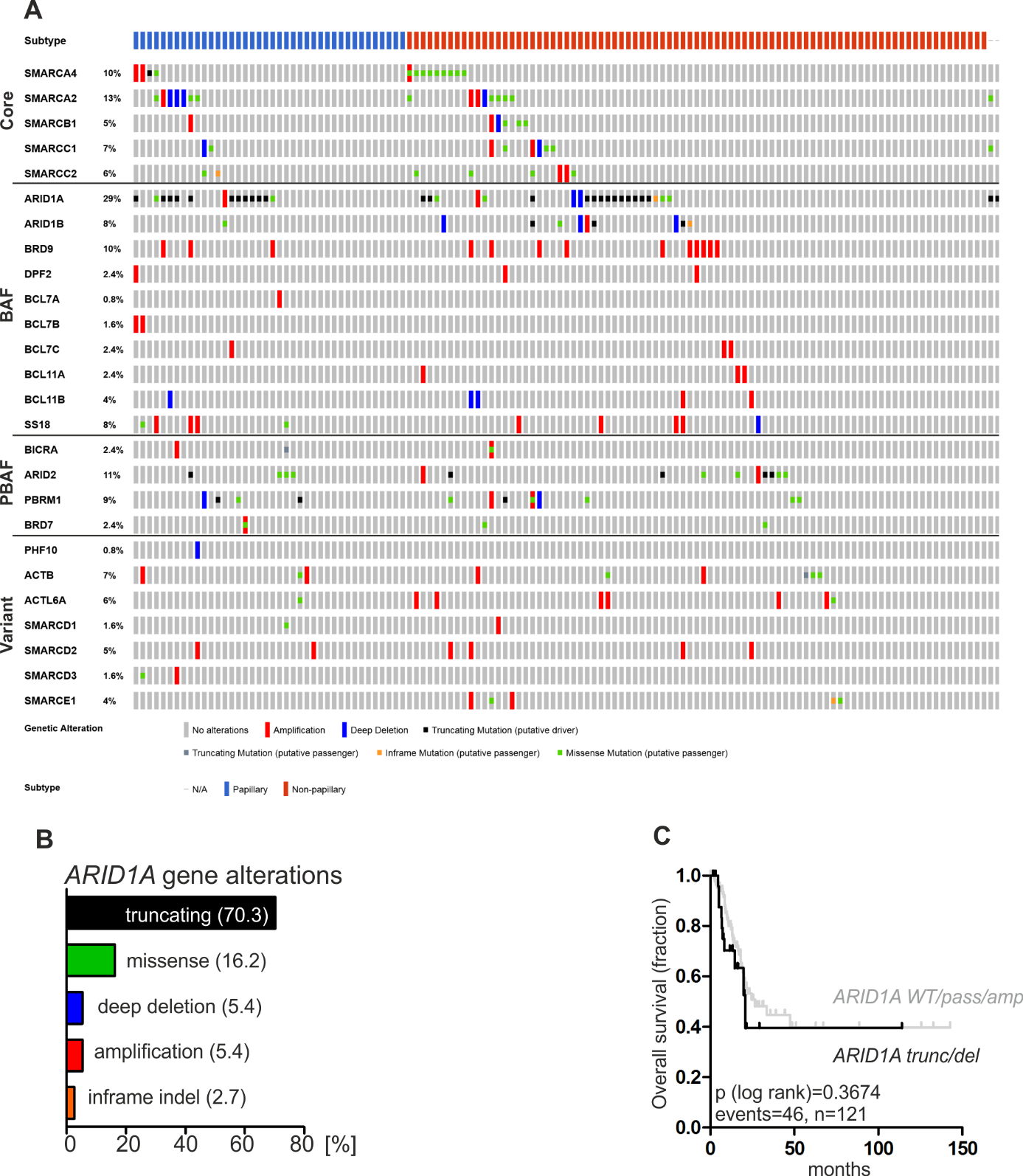
**

**S2 Fig. SWI/SNF subunit gene alterations in the TCGA 2014 data set.** (**A**) SWI/SNF subunit gene alterations including *ARID1A* are present in 93 cases (73%) of a TCGA cohort comprising 127 muscle-invasive urothelial bladder carcinomas with 37 patients (29%) showing *ARID1A* gene alterations. The 25 depicted subunit genes are categorized into „core“, „BAF-specific (BAF)“, „PBAF-specific (PBAF)“ and „variant/accessory (variant)“ subunit genes. Note: nBAF (neuronal BAF)-specific subunit genes have been excluded. (**B**) Frequencies of *ARID1A* gene alteration types in the TCGA cohort. (**C**) Univariate Kaplan-Meier survival curves displaying overall survival of patients harboring *ARID1A* truncating mutations (trunc) and deep deletions (del) (n= 28; black curve) in relation to patients with *ARID1A* putative passenger missense mutations (pass), gene amplification (amp) and wildtype (WT) *ARID1A* gene sequence (n= 93; grey curve).
