# Supplementary material for: ARID1A-deficiency in urothelial bladder cancer: No predictive biomarker for EZH2-inhibitor treatment response?
Source: PLoS One. 2018 Aug 23;13(8):e0202965. doi: 10.1371/journal.pone.0202965 (PMC6107234; doi:10.1371/journal.pone.0202965)
Supplement: S3 Fig — (DOCX) [file pone.0202965.s003.docx]

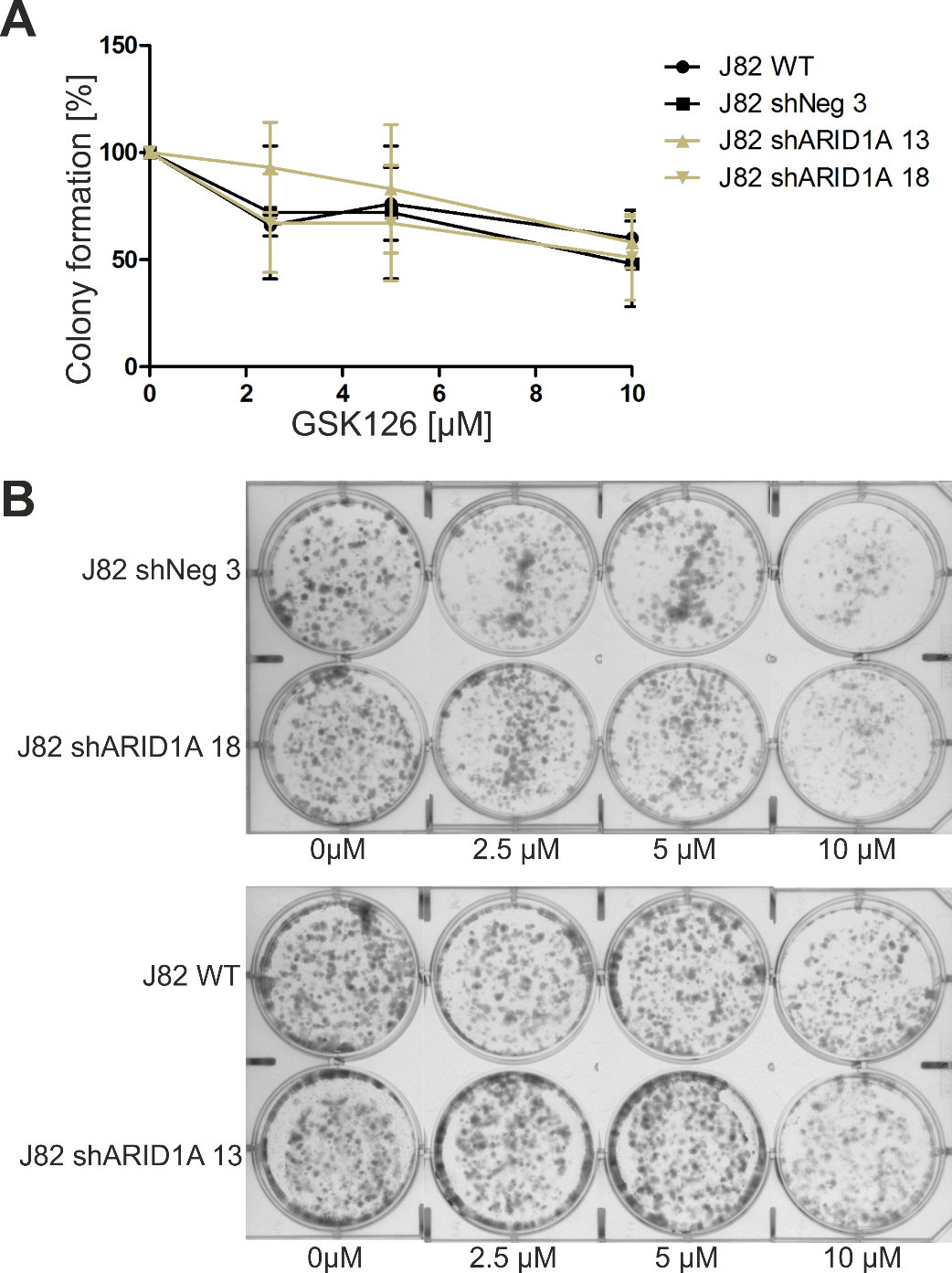


**S3 Fig. Long-term GSK126 treatment of J82 cells.** ARID1A-depleted J82 cells (J82 shARID1A 13 and 18) and controls (J82 WT and J82 shNeg 3) were exposed to different concentrations of GSK126 for 10 days. Afterwards colony formation was assessed densitometrically. (**A**) Mean colony formation at the indicated drug doses of three independent experiments is depicted. Error bars: Standard deviation, WT: wildtype. (**B**) Representative colony formation ability of J82 cells after GSK126 treatment for 10 days at the indicated drug doses.
