# Supplementary material for: ARID1A-deficiency in urothelial bladder cancer: No predictive biomarker for EZH2-inhibitor treatment response?
Source: PLoS One. 2018 Aug 23;13(8):e0202965. doi: 10.1371/journal.pone.0202965 (PMC6107234; doi:10.1371/journal.pone.0202965)
Supplement: S4 Fig — (DOCX) [file pone.0202965.s004.docx]

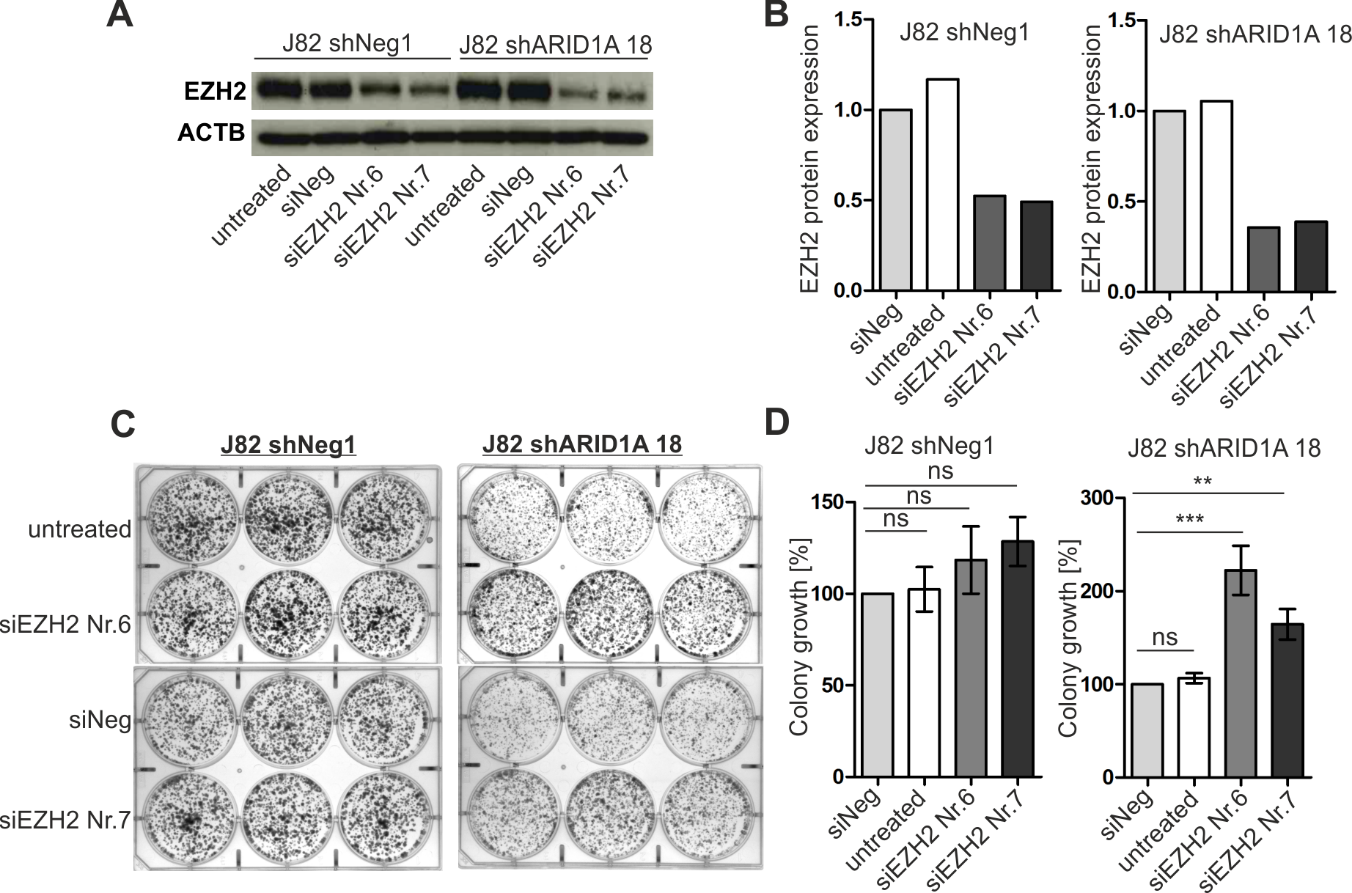


**S4 Fig. EZH2 protein depletion enhances colony growth of ARID1A-deficient J82 bladder cancer cells.** (**A**) Representative western blot result: EZH2 protein expression in an ARID1A-depleted J82 single-cell clone (J82 shARID1A 18) and a control vector-transfected J82 single-cell clone (J82 shNeg1) treated with two different *EZH2*-specific siRNAs (si*EZH2_6 and 7*) and a scrambled siRNA control (siNeg). Cells treated with transfection reagent only (untreated) were used as a second control. ACTB served as a loading control for western blot analysis. (**B**) Densitometrical evaluation of the western blot results shown in A. EZH2 protein expression level of scrambled siRNA (siNeg) treated cells of each clone was set to 1. (**C**) Representative colony formation assay in six-well plates containing cells of both single-cell clones treated with *EZH2*-specific siRNAs (si*EZH2_6 and 7*) in comparison to both controls (siNeg and untreated) two weeks after cell seeding. (**D**) Densitometrical evaluation of 2D colony growth of three independent experiments. The colony growth of the scrambled siRNA treated cells (siNeg) of each clone was set to 100%. Horizontal lines: mean values of triplicate experiments. Vertical lines: standard error of margin (SEM). Raw densitometrical data were used for statistical evaluation ** P <0.01, *** P <0.001, ns: not significant (Repeated measures ANOVA, Tukey’s multiple comparison test).
