# Supplementary material for: ARID1A-deficiency in urothelial bladder cancer: No predictive biomarker for EZH2-inhibitor treatment response?
Source: PLoS One. 2018 Aug 23;13(8):e0202965. doi: 10.1371/journal.pone.0202965 (PMC6107234; doi:10.1371/journal.pone.0202965)
Supplement: S5 Fig — (DOCX) [file pone.0202965.s005.docx]

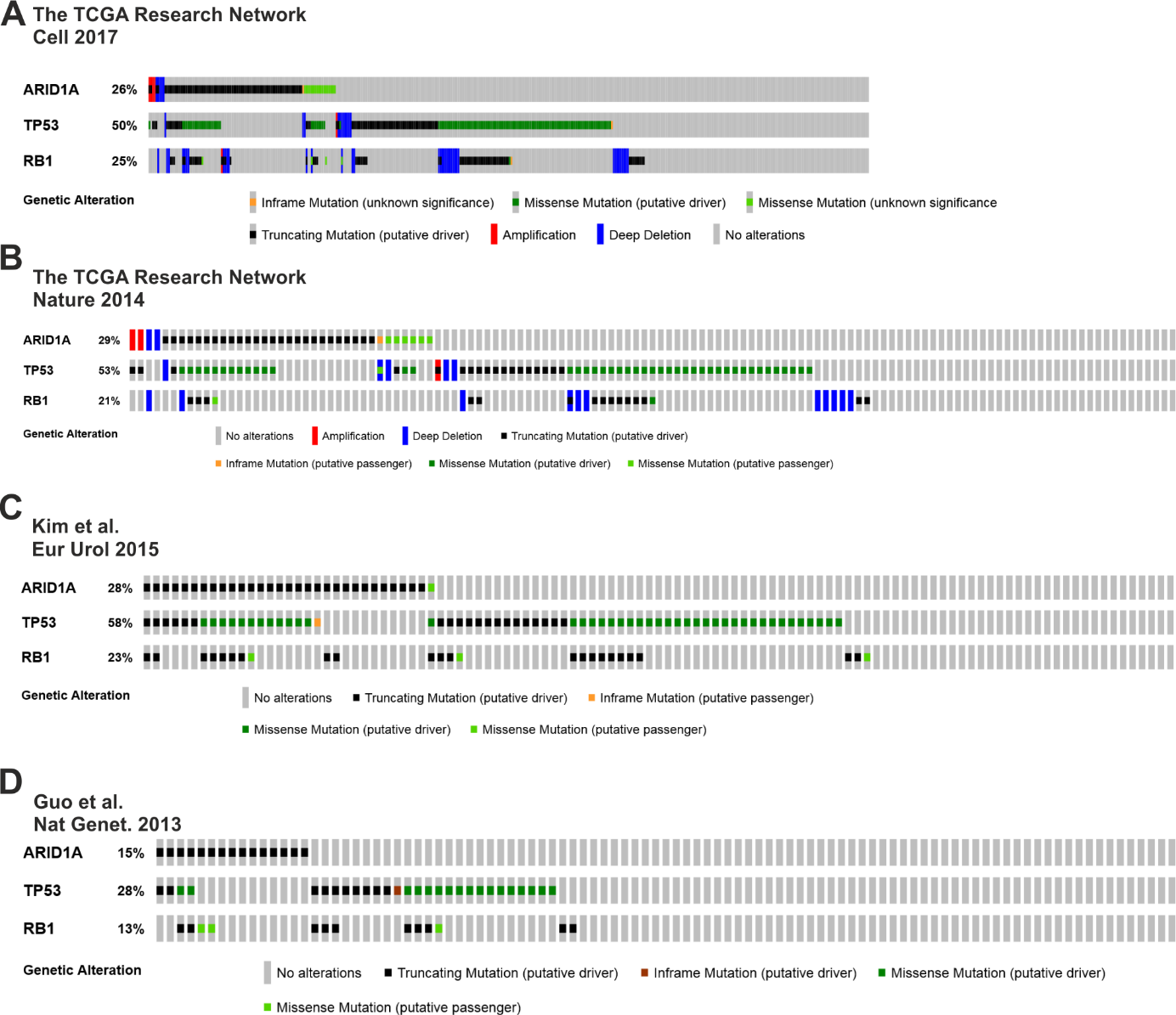


**S5 Fig.** **Genetic alterations (mutations and copy number variations) in *ARID1A* as well as *TP53* and *RB1*, two key genes driving urothelial bladder carcinogenesis.** The alteration frequencies of the mentioned genes in four independent bladder cancer sequencing studies [23,24,26,27] are depicted in **A**-**D**. OncoPrint (cBioPortal) was used for data visualization [25].
