# Supplementary material for: ARID1A-deficiency in urothelial bladder cancer: No predictive biomarker for EZH2-inhibitor treatment response?
Source: PLoS One. 2018 Aug 23;13(8):e0202965. doi: 10.1371/journal.pone.0202965 (PMC6107234; doi:10.1371/journal.pone.0202965)
Supplement: S6 Fig — (DOCX) [file pone.0202965.s006.docx]

**S6 Fig.** mRNA expression levels of key cell cycle-related genes (*MYC*, *CDKN1A*, *CCND1*), previously associated with *ARID1A* gene function, in TERT-immortalized normal human urothelial cells (TERT-NHUC) treated with an *ARID1A*-specific (siRNA_4) and control (siNeg) siRNA. The expression levels of the controls were set to 1. Vertical lines: ± standard error of margin (SEM).
