# Supplementary material for: ARID1A-deficiency in urothelial bladder cancer: No predictive biomarker for EZH2-inhibitor treatment response?
Source: PLoS One. 2018 Aug 23;13(8):e0202965. doi: 10.1371/journal.pone.0202965 (PMC6107234; doi:10.1371/journal.pone.0202965)
Supplement: S7 Fig — (DOCX) [file pone.0202965.s007.docx]

***CDKN1A***

***CCND1***

***MYC***

**S7 Fig.** mRNA expression levels of key cell cycle-related genes (*MYC*, *CDKN1A*, *CCND1*) previously associated with *ARID1A* gene function in UROtsa cells treated with an *ARID1A*-specific (siRNA_4) and control (siNeg) siRNA. The expression levels of the controls were set to 1. Vertical lines: ± standard error of margin (SEM).
