# Supplementary material for: ARID1A-deficiency in urothelial bladder cancer: No predictive biomarker for EZH2-inhibitor treatment response?
Source: PLoS One. 2018 Aug 23;13(8):e0202965. doi: 10.1371/journal.pone.0202965 (PMC6107234; doi:10.1371/journal.pone.0202965)
Supplement: S1 Table — (DOC) [file pone.0202965.s008.doc]

| **S1 Table. Primer sequences and PCR conditions for RNA expression analyses.** | | |
| --- | --- | --- |
| **Primer** | **Sequence** | **Product size [bp]** |
|
| ***ARID1A*  forward** | 5’-CCCCTCAATGACCTCCAGTA-3’ | 159 |
| ***ARID1A*  reverse** | 5’-CTGGAAATCCCTGATGTGCT-3’ |  |
|  |  |  |
| ***CCND1 forward*** | 5’-ACAGATCATCCGCAAACACG-3’ | 161 |
| ***CCND1 reverse*** | 5’-GGCGGTAGTAGGACAGGAAG-3’ |  |
|  |  |  |
| ***CDKN1A forward*** | 5’-ACTCTCAGGGTCGAAAACGG-3’ | 152 |
| ***CDKN1A reverse*** | 5’-AAGATGTAGAGCGGGCCTTT-3’ |  |
|  |  |  |
| ***MYC forward*** | 5’-GCTGCTTAGACGCTGGATTT-3’ | 142 |
| ***MYC reverse*** | 5’-CCTCCTCGTCGCAGTAGAAA-3’ |  |
|  |  |  |
| ***GAPDH* forward** | 5’-GAAGGTGAAGGTCGGAGTCA-3’ | 289 |
| ***GAPDH* reverse** | 5’-TGGACTCCACGACGTACTCA-3’ |  |
|  | | |
| **Real-time PCR reaction volumes of 20 µl consisted of the following components:** | | |
| 5 µM forward primer, 5 µM reverse primer, 10 µl SYBR GRN Supermix and 1 µl of cDNA as PCR template. Cycle conditions: 95°C for 3 min, 40 cycles of 95°C for 30 s, 60°C for 20 s, 72°C for 30 s. bp: base pairs. | | |
|
|
