# Supplementary material for: ARID1A-deficiency in urothelial bladder cancer: No predictive biomarker for EZH2-inhibitor treatment response?
Source: PLoS One. 2018 Aug 23;13(8):e0202965. doi: 10.1371/journal.pone.0202965 (PMC6107234; doi:10.1371/journal.pone.0202965)
Supplement: S2 Table — (DOCX) [file pone.0202965.s009.docx]

| **SWI/SNF complex** |
| --- |
| *SMARCA4*  *SMARCA2*  *SMARCB1*  *SMARCC1*  *SMARCC2*  *ARID1A*  *ARID1B*  *BRD9*  *DPF2*  *BCL7A*  *BCL7B*  *BCL7C*  *BCL11A*  *BCL11B*  *SS18*  *ARID2*  *PBRM1*  *BRD7*  *PHF10*  *ACTB*  *ACTL6A*  *SMARCD1*  *SMARCD2*  *SMARCD3*  *SMARCE1* |
| **PRC1/PRC2 complex** |
| *PCGF2*  *BMI1*  *PHC1*  *PHC2*  *PHC3*  *RING1*  *RNF2*  *CBX1*  *EZH2*  *EZH1*  *SUZ12*  *EED* |
| **RAS pathway genes** |
| *KRAS*  *NRAS*  *HRAS*  *BRAF* |

**S2 Table. Subunit genes of the SWI/SNF, PRC1/PRC2 complexes and Ras pathway genes analyzed in the cell lines J82 and RT112 for genetic alterations.**
