# Supplementary material for: ARID1A-deficiency in urothelial bladder cancer: No predictive biomarker for EZH2-inhibitor treatment response?
Source: PLoS One. 2018 Aug 23;13(8):e0202965. doi: 10.1371/journal.pone.0202965 (PMC6107234; doi:10.1371/journal.pone.0202965)
Supplement: S3 Table — (DOCX) [file pone.0202965.s010.docx]

**S3 Table.** Mutual exclusivity and co-occurrence analysis for *ARID1A*, *TP53* and *RB1* using sequencing data of the TCGA 2017 [23], TCGA 2014 [24], the Eur Urol 2015 [27] and the Nat Genet 2013 study [26]. cBioPortal was used to perform the analysis [25].

| **Gene A** | **Gene B** | **p-Value** | **Log Odds Ratio** | | **Association** |
| --- | --- | --- | --- | --- | --- |
| **TCGA 2017** | | | | | |
| *TP53* | *RB1* | <0.001 | 1.334 | Co-occurence | |
| *ARID1A* | *RB1* | 0.177 | 0.269 | Tendency towards co-occurrence | |
| *ARID1A* | *TP53* | 0.182 | -0.231 | Tendency towards mutual exclusivity | |
| **TCGA 2014** | | | | | |
| *TP53* | *RB1* | 0.031 | 0.945 | | Co-occurrence |
| *ARID1A* | *RB1* | 0.261 | -0.453 | | Tendency towards mutual exclusivity |
| *ARID1A* | *TP53* | 0.351 | 0.227 | | Tendency towards co-occurrence |
| **Eur Urol 2015** | | | | | |
| *TP53* | *RB1* | 0.009 | 1.339 | | Co-occurrence |
| *ARID1A* | *RB1* | 0.046 | 0.922 | | Tendency towards co-occurrence |
| *ARID1A* | *TP53* | 0.249 | 0.392 | | Tendency towards co-occurrence |
| **Nat Genet 2013** | | | | | |
| *TP53* | *RB1* | 0.001 | 2.071 | | Co-occurrence |
| *ARID1A* | *RB1* | 0.107 | 1.109 | | Tendency towards co-occurrence |
| *ARID1A* | *TP53* | 0.576 | -0.095 | | Tendency towards mutual exclusivity |
